# Dietary climate impact correlates ambiguously with health biomarkers– a randomised controlled trial in healthy Finnish adults

**DOI:** 10.1007/s00394-025-03609-w

**Published:** 2025-02-18

**Authors:** Merja Saarinen, Tiina Pellinen, Joel Kostensalo, Jouni Nousiainen, Katri Joensuu, Suvi T. Itkonen, Anne-Maria Pajari

**Affiliations:** 1 Natural Resources Institute Finland (Luke), Tietotie 4, Jokioinen FI-31600 Finland; 2 Department of Food and Nutrition, P.O. Box 66, University of Helsinki, FI-00014 Finland; 3 Natural Resources Institute Finland (Luke), Yliopistokatu 6B, Joensuu FI-80100 Finland; 4 Natural Resources Institute Finland (Luke), Latokartanonkaari 9, Helsinki FI FI-00790 Finland

**Keywords:** Dietary change, Sustainable diet, Food transition, Climate impact, Plant protein, Animal protein

## Abstract

**Purpose:**

A transition to more plant-rich diets is an effective way to reduce the climate impact of a diet. Using a whole-diet approach, we studied how partial replacement of animal-sourced with plant-sourced proteins affected the dietary climate impact while simultaneously considering diet-related health biomarkers.

**Methods:**

In a 12-week randomised controlled trial, 107 women and 29 men were assigned into three diet groups (ANIMAL, 50/50, PLANT) with animal-to-plant-protein ratios of 70/30, 50/50, and 30/70, respectively. Life-cycle-assessment-based coefficients for foods were used to assess the climate impact of the diet groups, based on four-day food records. Correlations between climate impact and biomarkers were assessed.

**Results:**

The climate impact (CO_2_ eq.) for PLANT was 3.32 kg per day, 3.05 kg per 2,000 kcal, and 0.04 kg per gram of protein, for 50/50 4.34, 4.20, and 0.05 kg, and for ANIMAL 4.93, 4.94, and 0.06 kg, respectively (*p* < 0.05 for all except ANIMAL vs. 50/50 /g protein and /2,000 kcal). Climate impact correlated weakly positively with colorectal cancer risk markers and a positive status of bone turnover, but not with cardiometabolic risk markers. Animal-based iron intake and climate impact (per 2,000 kcal) had a strong positive correlation 0.70 C.I. [0.60, 0.77], while saturated fat (0.29 [0.13, 0.44]) and calcium (0.37, [0.22, 0.51]) intake had a weak positive correlation, and fibre intake (− 0.37, [− 0.51, − 0.21]) a weak negative correlation with climate impact.

**Conclusion:**

Replacing animal-sourced proteins with plant-sourced proteins reduced the climate impact of the diet. The relationship between climate impact and biomarkers was more ambiguous indicated by both beneficial and harmful indicators within lower climate impacts.

**Clinical trial registry:**

NCT03206827; registration date: 2017–06–30.

**Supplementary Information:**

The online version contains supplementary material available at 10.1007/s00394-025-03609-w.

## Introduction

A transition to significantly more plant-rich diets was identified as one of the most important ways to reduce the climate impact of food consumption in Western countries in the first decade of the 21st century [1–3]. Plant-based diets have also been associated with nutritional and health benefits in both epidemiological [4–9] and modelling-based sustainability studies [10–12]. These health benefits include, e.g., lower risk of chronic diseases such as obesity, type 2 diabetes, CVD, and certain cancers, which may be a consequence of higher intakes of nutritionally beneficial components such as fibre, or lower contents of harmful substances such as saturated fat [13, 14]. On the other hand, relatively low intakes of some key nutrients from predominantly plant-based diets, such as vegan diets, have also been reported [15–17]. For example, plant-based diets may lead to lower intakes of key nutrients that are instrumental for, e.g., bone health, such as vitamin D and calcium [14, 18].

In recent years, there has been a strong emphasis on achieving environmental and nutritional benefits simultaneously, given that a transition to a more sustainable lifestyle is widely considered necessary and urgent [e.g., 19, 20]. Recently, the EAT-Lancet Commission released the Planetary Health Diet, a global reference diet, with the goal of promoting both health and climate benefits [21]. It presents recommendations of a reasonable consumption range for several basic food ingredients. As the model has been designed for global use, the report emphasises that the dietary recommendations need to be interpreted and adapted locally to reflect the culture, geography, and demographics of the population and individuals [21].

Animal-sourced food products are currently the predominant protein source in Western diets [22, 23]. It has been well established that plant protein sources such as legumes, cereals, seeds, and nuts tend to have a lower climate impact per unit mass than animal-based products [24–26]. The direct consumption of plant-sourced proteins rather than recycling them through domestic animals for human consumption is, therefore, a key strategy in moving towards a more sustainable diet.

The climate benefits obtained by replacing animal-sourced proteins with plant-based ones have been the subject of considerable sustainability research in recent years [15, 27, 28]. Modelling studies are usually based on healthy diet scenarios, linear optimisation of nutrition and climate, or wider environmental impacts, typically with an aim to identify an adequate or ideal sustainable diet, while population-based studies provide data on realised diets. However, little attention has been previously paid to the change dynamics [29], changes in the whole diet, and related climate impacts as protein sources change [30], as well as the relationship between health biomarkers and dietary change. A change in protein sources can lead to wider changes in diet or eating habits [31; 32], which in turn may affect the climate impact of a given diet or have nutritional and health consequences in a broader sense.

In this study, we used a whole diet approach in a randomised controlled trial to evaluate how the partial replacement of animal-based proteins with plant-sourced proteins affects the estimated climate impact of the diet, considering that the change in the protein source also alters the composition of the diet in other aspects. We assessed the climate impacts of the intervention diets and cross-sectionally investigated correlations between climate impact and several health indicators and nutrients at the end of the trial. The effects of partial replacement of animal-sourced proteins with plant-sourced proteins on nutrient intakes, nutritional status, and risk factors of chronic diseases in the trial have been previously studied and published elsewhere [33–35]. We also analysed the variability of climate impacts within the intervention groups. To our knowledge, this is the first randomised controlled trial (RCT) to combine climate impact with diet and health biomarkers.

## Methods

### Study population and food consumption

This study was part of a 12-week randomised controlled trial specifically designed to study the nutritional and health effects of partial replacement of dietary animal-sourced proteins with plant-sourced proteins. The study was approved by the Coordinating Ethics Committee of the Hospital District of Helsinki and Uusimaa (1651/2016), and all participants provided their written informed consent before the first research visit. The implementation of the trial is described in detail by Päivärinta et al. [33].

The participants (*n* = 136) comprised of 107 women and 29 men (20–69 years). The participants were randomised into three groups with a different dietary protein composition: (1) ANIMAL, animal-sourced protein 70%/plant-sourced protein 30% of total protein intake (2) 50/50, animal-sourced protein 50%/plant-sourced protein 50% and (3) PLANT, animal-sourced protein 30%/plant-sourced protein 70%. The targeted protein intake in all diets was 17% of energy intake (E%). The ANIMAL diet corresponded to the average Finnish diet in terms of proportions of animal- and plant-source proteins [36]. In the 50/50 diet, the consumption of red meat did not exceed the recommended maximum of the national nutrition recommendations (500 g cooked weight/week) [37]. Food consumption data prior to intervention and during the last week of the intervention were collected using four-day food records and analysed by AivoDiet software (version 2.2.0.1, Aivo Oy, Turku, Finland), including the Fineli^®^ Food Composition Database Release 16 (2013), maintained by the Finnish Institute for Health and Welfare [38], and is described in more detail in [33]. The software provided data on individual diets (*n* = 136), on nutrient intakes, and specifically on food items and dishes, as well as the amounts consumed, which were used in the climate impact assessment of the diets (ANIMAL *n* = 46, 50/50 *n* = 46, PLANT *n* = 44).

The energy content of the intervention diets was based on the average energy consumption of 8,400 kJ/d (2,000 kcal/d), but the participants were advised to maintain a stable weight and eat according to their appetite. The participants were provided with most of their protein sources to be consumed at home by the study: these included meat and meat products, poultry products, fish and vegetable patties, ready-made meals, frozen or dried pulses and other legume-based products, nuts, seeds, bread, and cereals, and excluded dairy products and eggs, which the participants were advised to use according to the instructions provided (Supplement Table A1). Based on the food records, the controlled food items in the intervention diets supplied an average of 80% of the daily energy intake [33].

The participants were allowed to consume habitual amounts of foods with a low protein content, such as fruit, vegetables, juices, confectioneries, and alcoholic beverages. The greatest flexibility was thus in the consumption of products other than protein sources, though some flexibility was also allowed in protein sources; for example, a product provided by the study was allowed to be replaced with an equal amount of a comparable product when, e.g. eating out at a restaurant. However, according to the instructions, the subjects had to keep the consumption of beef, for example, equal to the amount distributed in the experiment, and not replace it with another type of meat. As the participants were given both foods to be eaten as such, as well as food ingredients, they were instructed to implement the diet at a food level, and some recipes were also delivered to support the implementation especially regarding the use of plant-protein products. In addition, when adjusting the total quantity of food or energy to suit themselves, the participants were instructed to keep the ratio of different product groups unchanged. Due to the nature of the intervention, total blinding (research staff or participants) was not possible, and color codes were used to mark the diets.

### Assessment of the climate impact

The climate impact of the diets was assessed by combining life cycle assessment (LCA) based global warming potential (GWP) coefficients for products, food items, and dishes contained in the diets of the participants using SAS software (version 9.4). The diets included 3,075 products in total. The products were classified in 107 product groups for which the GWP coefficients were estimated based on the literature and LCA studies for Finnish agricultural and food products conducted previously by Natural Resources Institute Finland. For products for which coefficients were missing in these data sources, we used approximations based on LCA-estimates of similar products. For dishes, coefficients were based on typical Finnish industrial and homemade recipes. Each product group included a variety of products, and therefore the product groups were given estimates with a minimum and maximum value, the average of which was used as the coefficient to calculate the climate impact of the diets. The data for determining the average GWP coefficients of food items were chosen to correspond to the food consumed in Finland as well as possible. The final coefficients for the product groups, as well as related data sources, are shown in Supplement Table B1.

Most of the data used followed system boundaries that included life cycle phases from agriculture and production of its inputs to industrial manufacturing, transport and packaging, retail and home cooking, and cold storage. However, the original data on many product systems did not include some phases, but these were added by using data presented in Supplement Tables B1 and B2. The main assumptions in the calculation of the product group-specific coefficients based on source data are also described in Supplement B.

In the assessment of the dietary climate impact, we first calculated the daily dietary climate impact for each person in the three diet groups, using three functional units (FU): daily intake; 2,000 kcal, and a gram of protein. Daily intake as an FU means the inclusion of total daily food intake in the assessment as such. Using 2,000 kcal as an FU is based on scaling each diet to correspond to 2,000 kcal of energy intake from the diet. Thus, this changes the amount of food in personal diets, or the reference flow in LCA terminology, compared to the realised diets. As the study addresses proteins, we also selected a gram of protein as an FU, because it reveals the climate efficiency of a diet as a protein source. After calculating results for the individual diets, we calculated the group means to obtain average results for each intervention diet.

### Biomarkers and nutrients for risk of chronic diseases

We addressed main chronic diseases in Finland [39–41] in our analysis by using biomarkers and nutrient intake from the intervention. The endpoint data of the trial were used for the comparisons without separation to subgroups based on intervention grouping i.e. the analysis regarding these outcomes is cross-sectional. Blood lipid concentrations (non-HDL-cholesterol mmol/l, LDL/HDL ratio, triglycerides mmol/l), glucose homeostasis markers (insulin mU/l, glucose mmol/l, homeostatic model 2 assess insulin resistance; HOMA2-IR), bone turnover markers (the ratio of procollagen type I amino-terminal propeptide, PINP, ng/ml, and collagen type 1 cross-linked C-terminal telopeptide, CTX, ng/ml), and faecal water N-nitroso compounds (total and haem, pmol/mg faeces) were used as indicators of cardiovascular disease, type 2 diabetes, bone health, and colorectal cancer, respectively. These measures have been previously explored in [33–35, 42, 43]. In addition, body mass index (BMI) and blood pressure (mmHg) were of interest. Nutrients associated with these chronic diseases were included in the analysis: Intake of saturated fat was included as a cardiovascular health/risk factor, and vitamin D and calcium were analysed as having an impact on bone health, and animal-sourced iron and fibre having an impact on colorectal cancer risk. These intakes have been previously published [33–35].

### Statistical analysis

The primary outcome of the study was to compare climate impacts between the diet groups. Because there were several outliers in the climate impacts of the ANIMAL and 50/50 groups, and the subgroup data therefore failed to pass the Shapiro-Wilk normality test, between-group comparisons were carried out using a permutation test. Given that multiple comparisons between the groups are made, we took a conservative approach and adjusted the *p*-values using a Bonferroni correction for the between-group dietary comparisons. The secondary outcome, correlations between climate impacts (as such and per 2,000 kcal/d) and biomarkers of chronic disease risks as well as of chosen nutrient intakes were analysed with Spearman correlations and their 95% confidence intervals with all participants included using measurements from the endpoint of the intervention. The goal here was to find out which health biomarkers are strongly associated with the climate impact, and which were not. Level of relevant correlation was set at below *r* = − 0.3 or above *r* = + 0.3, and statistical significance at *p* < 0.05. Correlations between − 0.3 < *r* < + 0.3 were considered sufficiently weak, so that the health biomarker could be affected without necessarily reducing or increasing the climate impact, i.e. low or high biomarker values could be achieved with diets with high or low climate impact. Correlations with *r* < − 0.3 were interpreted as indicating that a dietary change resulting in an increase in the biomarker is very likely associated with a decrease in climate impact, and a correlation with *r* > 0.3 as indicating that a dietary change resulting in an increase in the biomarker is very likely associated with an increase in climate impact. Additionally, correlations were checked within intervention groups to reveal any between-group differences, but the sample sizes were of course much smaller for these analyses, and thus the statistical power was not sufficient to reliably observe correlations below|*r*|= 0.30, while correlations above|*r*|> 0.17 could be detected from the full sample. The insulin, HOMA2-IR, and bone turnover marker results were missing from two participants, and total and haem NOC marker results from one participant. In addition, two subjects were excluded from the bone turnover marker analyses due to severe hyperparathyroidism. The statistical tests were carried out using the statistical software R [43].

## Results

Characteristics of the participants (*n* = 136) are described on Supplemental Table C1. Mean age of the participants was 48 years, they were mostly women (*n* = 107) and highly educated (Supplemental Table C1).

### Dietary compliance

**Fig. 1:**
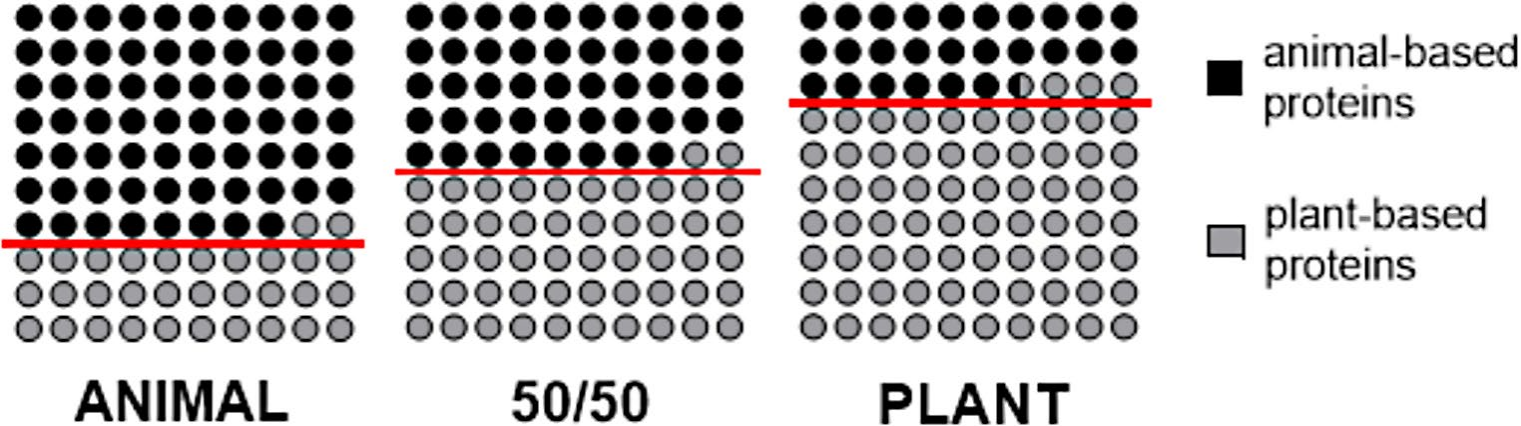
Planned and realised proportions of animal- and plant-source proteins in the intervention diets. The ANIMAL diet was planned to contain 70% of animal-source proteins and 30% of plant-source proteins, 50/50 diet equal amounts of animal- and plant-source proteins, and the PLANT diet 30% of animal- and 70% of plant-source proteins. Each grey circle represents 1% of the realised share of plant-source protein, and each black circle 1% of the realised share of animal-source protein. The planned shares of plant- and animal-source proteins in each diet are separated by the red line; the share of plant-source protein is below, and that of animal-source proteins above, the red line

The distribution of animal and plant-sourced proteins in the realised diets was as planned (Fig. 1). The intended protein intake of 17 E% was achieved in the ANIMAL (mean 18.2 E%, SD 3.1 E%) and 50/50 (mean = 16.9 E%, SD ± 2.2 E%) groups when the mean intakes were examined. In the PLANT group, the mean protein intake was lower (mean = 15.2 E%, SD ± 2.0 E%) than in the ANIMAL and 50/50 diet groups (*p* < 0.001 and *p* = 0.002 respectively). There were no differences between the groups in energy, carbohydrate, or fat intake at the end of the study [33].

### The climate impact of the diets at group level

**Fig. 2:**
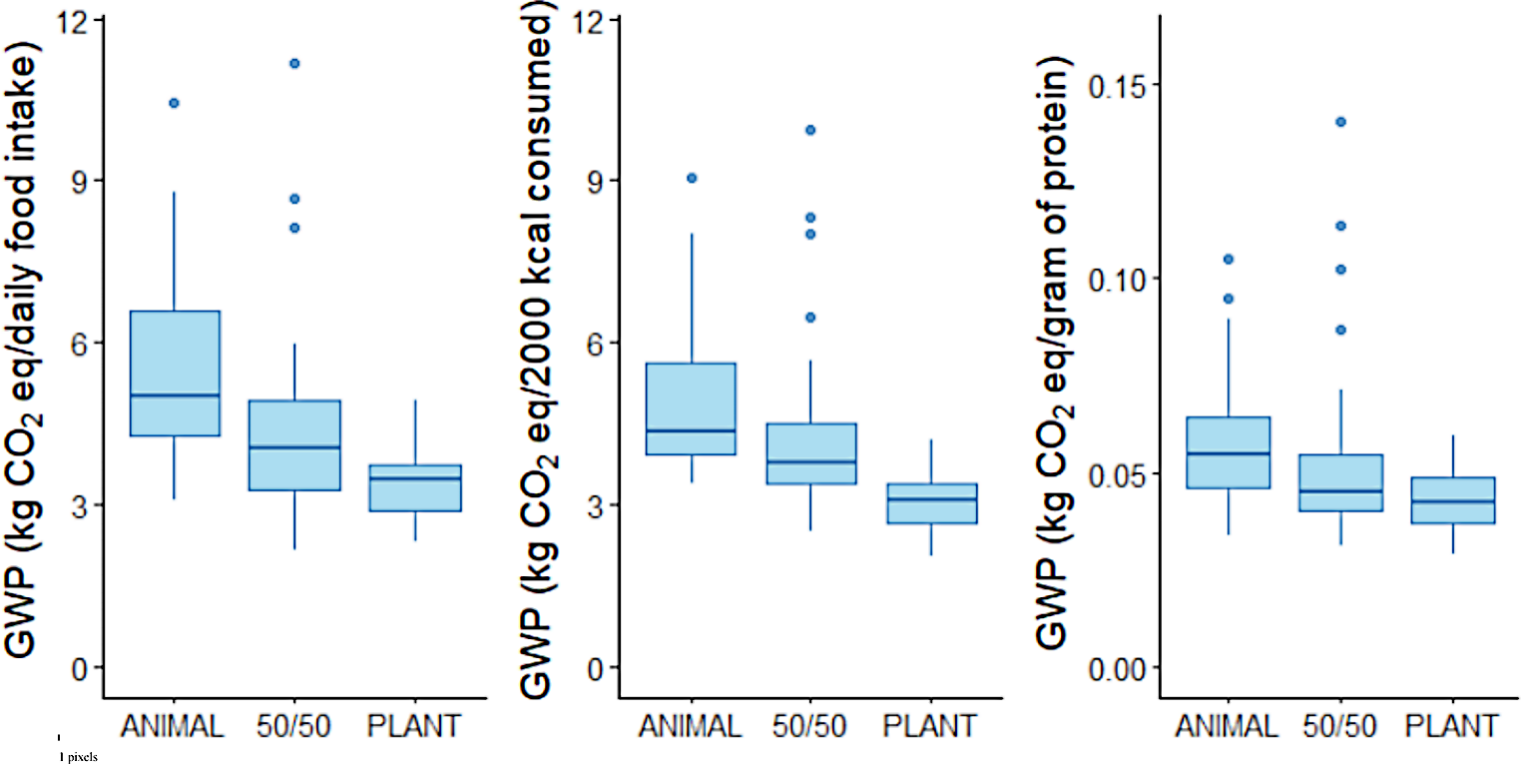
The climate impact as global warming potential (kg CO_2_ eq). of the intervention diets in the intervention groups ANIMAL, 50/50, and PLANT using three FUs: **a**) daily food intake; **b**) 2,000 kcal; and **c**) a gram of protein. The three intervention diets consisted of ANIMAL: 70% animal-source protein/30% plant-source protein; 50/50: 50% animal-source protein/50% plant-source protein; and PLANT: 30% animal-source protein/70% plant-source protein

The climate impact of the realised diet (daily food intake as an FU) was highest in the ANIMAL group and lowest in the PLANT group (Fig. 2; Table 1). The climate impact of the 50/50 diet was an average of 1.08 kg CO_2_ Eq. (20%) lower than in the ANIMAL diet (*p* = 0.03), and for PLANT diet, the impact was 2.10 kg CO_2_ eq. lower (39%, *p* < 0.001). The difference between the 50/50 and PLANT diets was also statistically significant (*p* = 0.005).

**Table 1:** Average climate impacts of the three intervention diets realised, 2,000 kcal/d adjusted, and protein-adjusted in kilograms of CO_2_-equivalent (kg CO_2_ eq.), their standard errors (SE), and standard deviations (SD). The three intervention diets were ANIMAL: 70% animal-source protein/30% plant-source protein of total protein intake; 50/50: 50% animal-source protein/50% plant-source protein; and PLANT: 30% animal-source protein/70% plant-source protein

	Diet	Mean climate impact (kg CO_2_ eq.)	SE (kg CO_2_ eq.)	SD (kg CO_2_ eq.)
Realised	Animal	5.42^a^	0.24	1.62
	50/50	4.34^b^	0.25	1.68
	Plant	3.32^c^	0.09	0.58
Per 2,000 kcal	Animal	4.94^a^	0.19	1.32
	50/50	4.20^a^	0.22	1.47
	Plant	3.05^b^	0.08	0.52
Per g protein	Animal	0.056^a^	0.002	0.015
	50/50	0.052^a^	0.003	0.021
	Plant	0.042^b^	0.001	0.008

^a, b,c)^ Groups with different letters for a given functional unit (realised, 2,000 kcal, g protein) are statistically significant at the *p* < 0.05 level after controlling for the family-wise error rate with a Bonferroni correction

Using 2,000 kcal or a gram of protein as the FU did not change the order of the diets with respect to the climate impact, although the differences were narrowed. When adjusted for 2,000 kcal, the differences were as follows: 50/50 vs. ANIMAL: -0.74 kg CO_2_ eq. (-15%, *p* = 0.12), PLANT vs. ANIMAL vs.: -1.89 kg CO_2_ eq. (-38%, *p* < 0.001), and PLANT vs. 50/50: -1.15 kg CO_2_ eq. (*p* < 0.001). We observed a similar pattern when a gram of protein was used as the FU. The differences were as follows: 50/50 vs. ANIMAL: -0.004 kg CO_2_ eq. (-7.1%, *p* > 0.99), PLANT vs. ANIMAL: -0.014 kg CO_2_ eq. (-25%, *p* < 0.001), and PLANT vs. 50/50: 0.010 kg CO_2_ eq. (*p* = 0.05).

### Climate impact and the biomarkers and nutrients

**Fig. 3:**
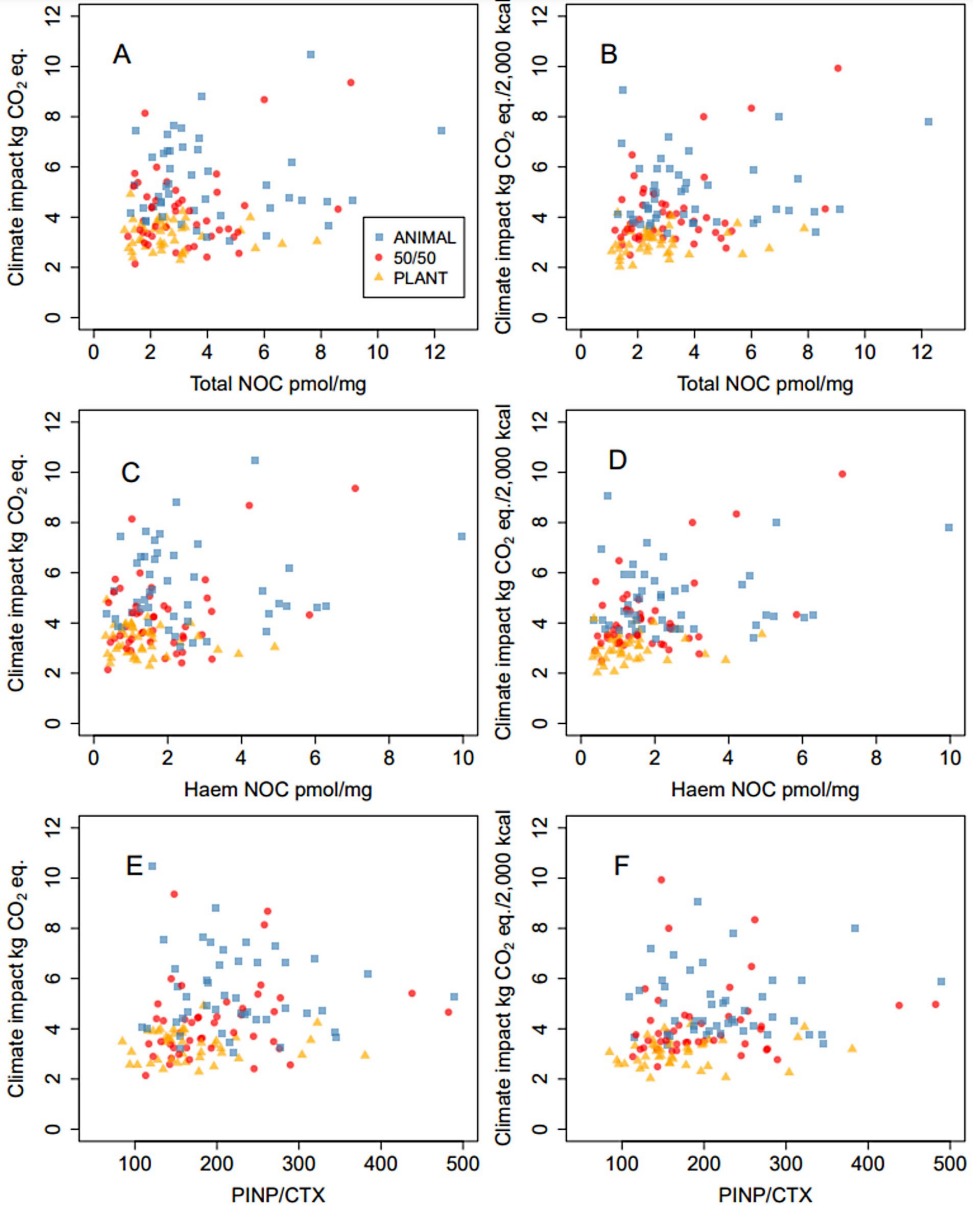
Scatter plots of **A**) climate impact kg CO_2_ eq. total NOC pmol/mg, **B**) climate impact kg CO_2_ eq./2,000 kcal and total NOC pmol/mg, **C**) climate impact kg CO_2_ eq. and haem NOC pmol/mg, **D**) climate impact kg CO_2_ eq./2,000 kcal and haem NOC pmol/mg **E**) climate impact kg CO_2_ eq. and PINP/CTX ratio, **F**) climate impact kg CO_2_ eq./2,000 kcal and PINP/CTX ratio. Blue rectangles = ANIMAL diet (70% animal-source protein/30% plant-source protein), red circles = 50/50 diet (50% animal-source protein/50% plant-source protein) and orange triangles = PLANT diet (30% animal-source protein/70% plant-source protein) participants. In subfigures 3 A–B *n* = 135, subfigures C–D *n* = 134, and figures E–F *n* = 132

**Fig. 4:**
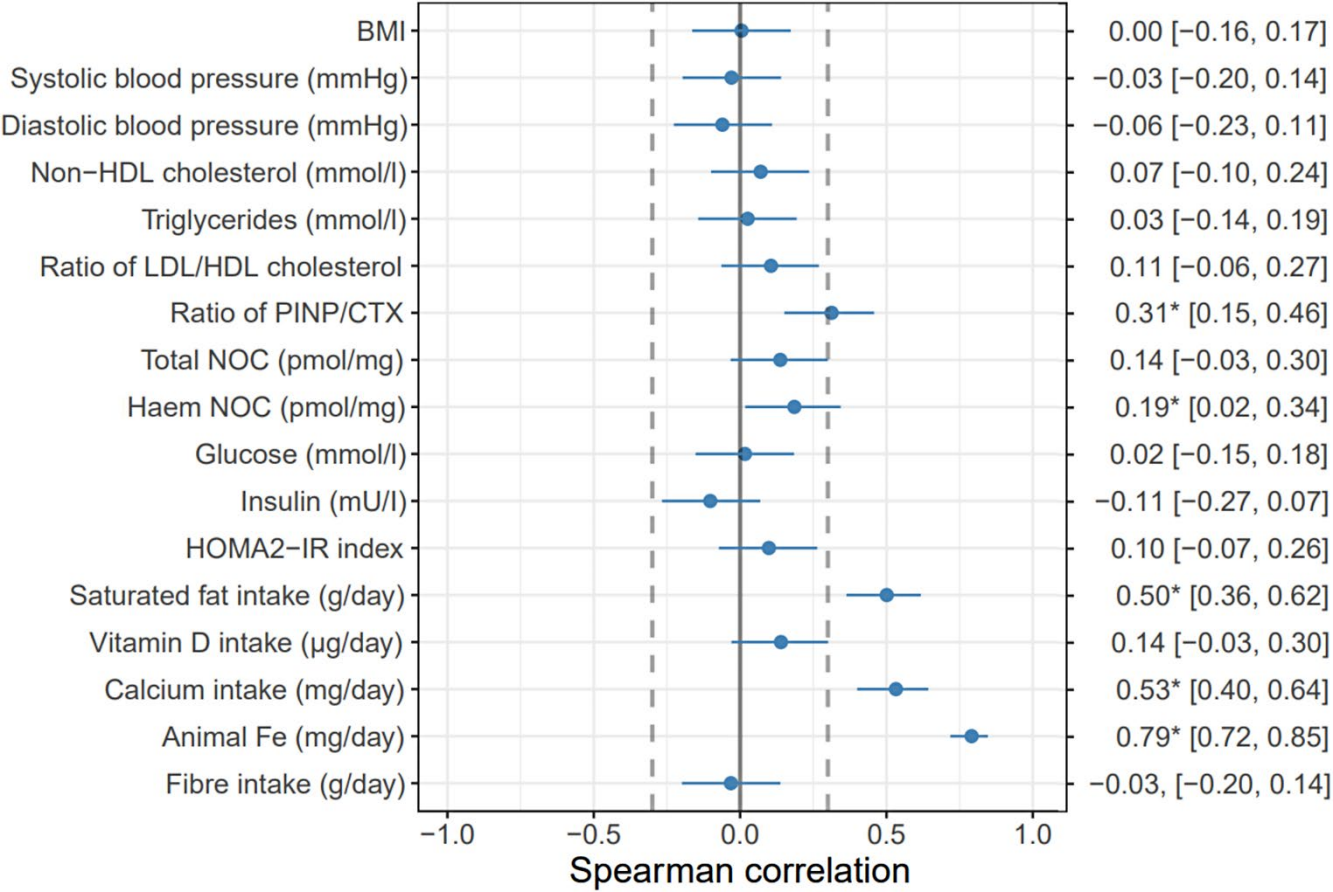
Spearman correlations and their 95% C.I. of climate impact (kg CO_2_ eq.) with health biomarkers after a 12-week intervention and intake of animal Fe, calcium, vitamin D, and saturated fat during the intervention. The dashed lines mark the limit of relevant correlation, set at below *r* = − 0.3 or above *r* = + 0.3. The correlations which are statistically significant at the *p* < 0.05-level are highlighted with an asterisk (*)

**Fig. 5:**
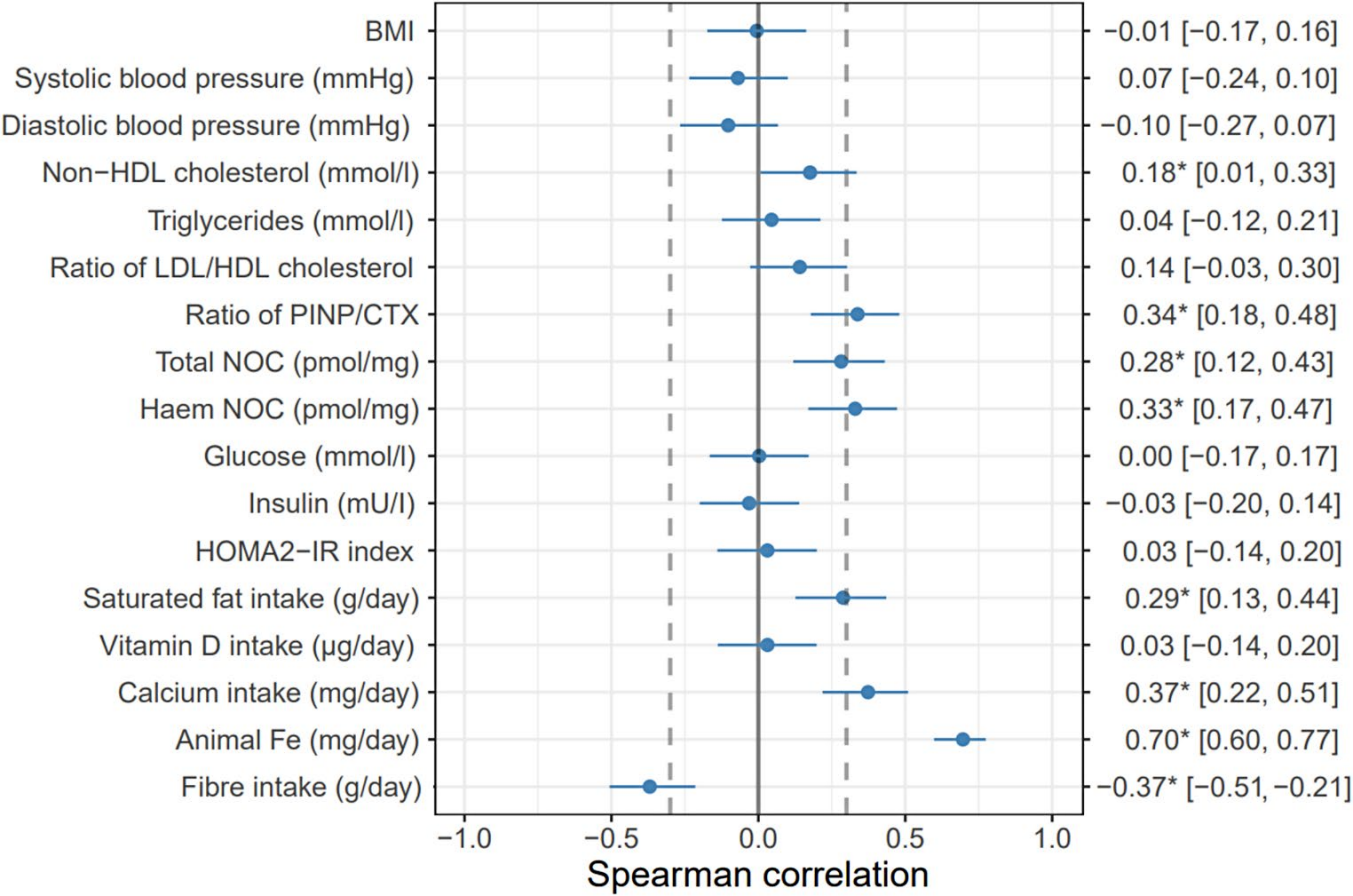
Spearman correlations and their 95% C.I. of climate impact (kg CO_2_ eq./2,000 kcal.) with health biomarkers after a 12-week intervention and intake of animal Fe, calcium, vitamin D, and saturated fat during the intervention. The dashed lines mark the limit of relevant correlation, set at below *r* = − 0.3 or above *r* = + 0.3. The correlations which are statistically significant at the *p* < 0.05-level are highlighted with an asterisk (*)

The Spearman correlations and their 95% C.I. of climate impact (kg CO_2_ eq.) with health biomarkers at the end of a 12-week intervention and intake of animal Fe, calcium, vitamin D, and saturated fat during the intervention are presented in Fig. 3 and per 2,000 kcal in Fig. 4. Total N-nitroso compound concentrations correlated positively with climate impact per 2,000 kcal (*r* = 0.28 C.I. [0.12, 0.43]) but not with climate impact of the diets as such (*r* = 0.14 [− 0.03, 0.30]). Haem N-nitroso compound correlated with climate impact per 2,000 kcal (*r* = 0.33 [0.17, 0.47]) and weakly with climate impact of the diets as such (*r* = 0.19 [0.02, 0.34]). (Fig. 5A–D). PINP/CTX ratio, indicating the ratio between bone formation and resorption also correlated positively with the climate impacts as such and per 2,000 kcal (*r* = 0.31 [0.15, 0.46] and *r* = 0.34 [0.18, 0.48], respectively) (Fig. 5E–F). In a group level, PINP/CTX ratio correlated positively with climate impact as such in 50/50 group (*r* = 0.32, *p* = 0.03) (Supplemental Table C1). Correlations between lipid concentrations, glucose metabolism markers, BMI, or blood pressure and climate impacts were not statistically significant (*p* > 0.05) when including all the participants (Figs. 3 and 4). In a group level analysis, LDL/HDL ratio, and non-HDL cholesterol correlated positively with climate impact per 2,000 kcal in the 50/50 group (*r* = 0.33, *p* = 0.02 and *r* = 0.31, *p* = 0.038), and HOMA2-IR index positively with climate impact as such in the ANIMAL group (*r* = 0.30, *p* = 0.05) (Supplemental Table C1).

Of the chosen nutrients, calcium, saturated fat, and animal-sourced iron intake correlated positively with the climate impact as such and per 2,000 kcal (*r* = 0.37 [0.22, 0.51], 0.29 [0.13, 0.44], and 0.70 [0.60, 0.77], respectively) whereas vitamin D intake did not show any correlation (*r* = 0.03 [− 0.14, 0.20]) (Figs. 3 and 4). When considering the groups separately, calcium intake correlated positively with climate impact as such in the ANIMAL and PLANT groups, vitamin D intake correlated negatively with climate impact per 2,000 kcal in the 50/50 group, and animal-sourced iron intake positively with both climate impact as such and per 2,000 kcal in every diet group (*r* = − 0.31–0.71, *p* = 0.04–<0.001). Fibre intake correlated positively with climate impact as such in the ANIMAL and 50/50 groups (*r* = 0.37, *p* = 0.01, and *r* = 0.33, *p* < 0.01), and negatively with climate impact per 2,000 kcal in the PLANT group (*r*=-0.44, *p* < 0.01) (Supplemental Table C1).

### Variability of the individual-level dietary climate impacts within the diet groups

**Fig. 6:**
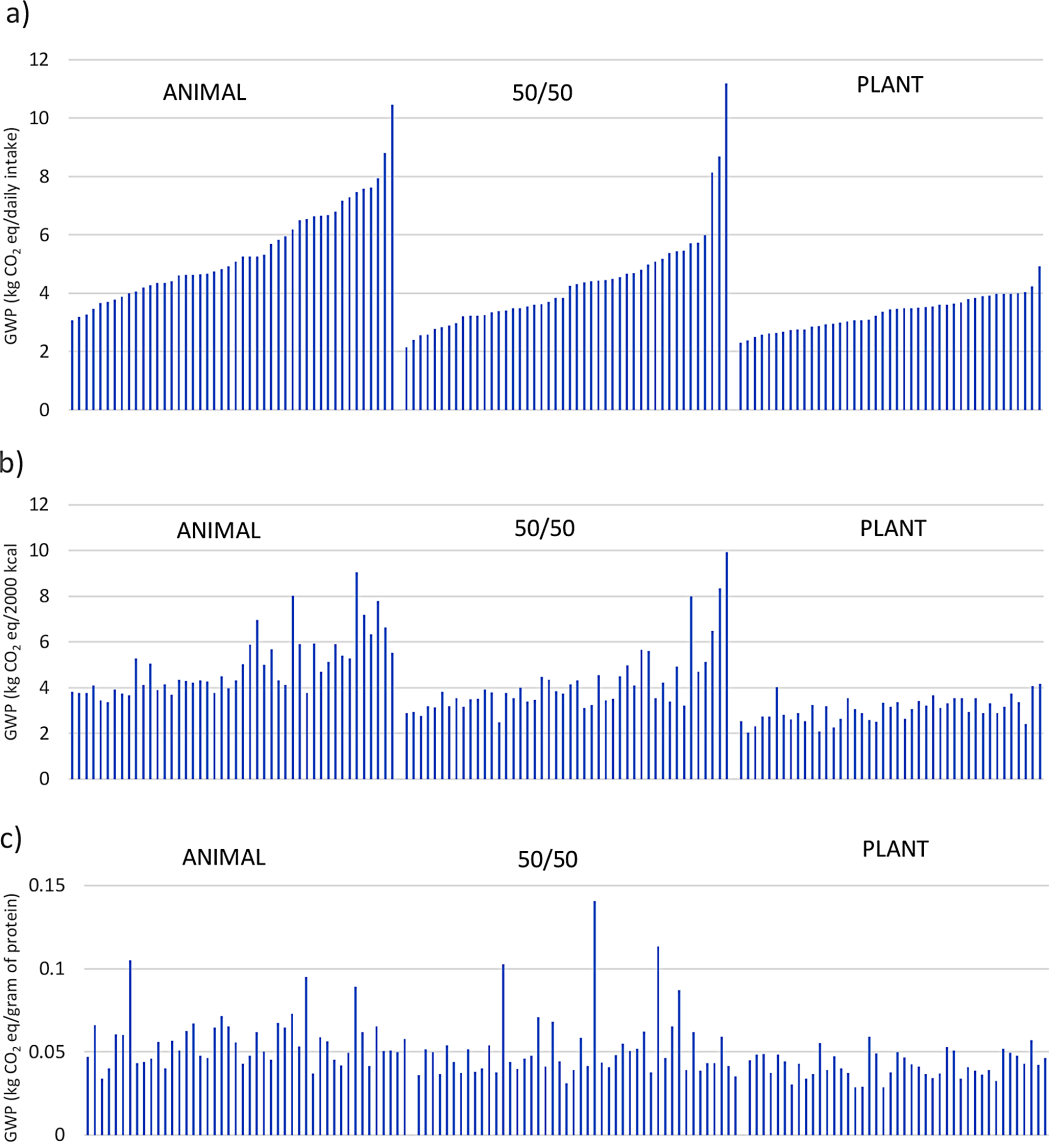
The climate impact of personal diets in the intervention groups ANIMAL, 50/50 and PLANT: **a**) realised diets (kg CO_2_ eq. per daily intake); **b**) energy-adjusted diets (kg CO_2_ eq. per 2,000 kcal); and **c**) protein intake (kg CO_2_ eq. per gram of protein)

There was large variability in the climate impacts of diets within the intervention groups, particularly in the 50/50 and ANIMAL groups (Table 1; Fig. 6a-c). However, when the results based on different FUs are compared, one can observe that the choice of FU considerably affects the results of individual diets. In Fig. 6a-c, the individual diets of the groups are arranged according to the climate impact of realised diets (6a).

## Discussion

Using a whole-diet approach, we evaluated here how the partial replacement of animal-sourced proteins with plant-sourced proteins affected the estimated climate impact of the diet, and whether there is a cross-sectional relationship between the climate impact and some health indicators. The study was carried out using data from a 12-week randomised controlled trial specifically designed to study the nutritional and health effects of a dose-dependent switch from animal to plant-based protein sources [33]. Our main result is that replacing animal-sourced proteins with plant-sourced proteins reduces the climate impact of the diet, meanwhile the relationship between climate impact and health biomarkers is more ambiguous indicated by both beneficial and harmful consequences within lower climate impact.

In the PLANT diet, where 70% of total protein intake originated from plant-sourced proteins, the climate impact was 25–38% smaller than the climate impact of the ANIMAL diet with 70% of animal-source proteins, depending on the comparison basis, i.e., the functional unit (FU) used. By contrast, the climate impact of the 50/50 diet with a similar amount of plant and animal-sourced proteins was 7–20% smaller than the climate impact of the ANIMAL diet, and the difference was statistically significant only regarding the realised diets. The PLANT diet would therefore be the best in terms of climate impact alone.

As is typical in LCA studies, using different FUs produced different results. The choice of FU is crucial, and according to the LCA methodology [46, 47], it should be in line with the goal and scope of the study; different FUs fit into different situations and answer different questions [46]. In general, when assessing the climate impact of diets, the standardisation is usually done for energy content, because it is considered a good basis for comparability. Our result (− 38%) for an *energy-adjusted* PLANT diet with a reduction of animal-source protein of 57% (ANIMAL vs. PLANT) is well in line with the estimates presented in the literature which have shown that the climate impacts of vegetarian diets can be 20–35% lower than those of current Western diets, and the climate impacts of vegan diets can be 25–55% lower [27]. Also, diets composed in line with the nutrition recommendations but containing less meat and more plant-based foods than current (Western) diets can achieve a 20–50% reduction in the climate impact [47]. However, our result is somewhat lower than the maximum reduction potential based on a global analysis [20] where a reduction of up to 85% of the climate impact of diet in high-income countries has been reported if more plant-rich diets were adopted. In contrast, our results (− 15%) for an energy-adjusted 50/50 diet did not achieve any of these results. However, the dietary change also was quite moderate in this group, with only a 28% reduction in animal-sourced proteins. The differences between the studies may also be due to the different dietary references used. For example, the average food consumption data used a global analysis [20] for high-income countries differed largely from the current Finnish diet [36], as did the climate impact coefficients for foods. However, different research objectives and methodological differences between the studies may also explain the differing results.

The results for *diets as such* are especially in line with the results from a study of the nutritionally adequate dietary scenarios for the whole Finnish population [48], in which halving meat intake reduced the climate impact of an average diet by 14% and reducing meat intake to a third by 20%. In that study, the intake of dairy products was not reduced, except for a slight decrease in cheese consumption. This suggests that dairy consumption is not as crucial for the climate impact of the diet as meat consumption. Interestingly, the results of these two studies on Finnish diets are in excellent agreement, although food grouping and the compilation of the corresponding climate impacts data utilised different approaches. This suggests that the LCA-based results are robust with respect to climate impact data. More generally, it may also suggest that LCA-based assessment results of dietary climate impacts may not be highly sensitive to the consistency of source data for products’ climate impact although strict criteria have been proposed to data quality of LCA data in dietary assessment [50]. However, this robustness should be further validated in the future research.

While between-group variation in climate impacts could be expected, the large within-group variation in climate impacts was surprising. When meat consumption in personal diets was examined more closely, it was found that the groups were somewhat mixed at the extremes: for example, based on meat consumption, the individuals in the 50/50 group who consumed the most meat would in fact have belonged to the ANIMAL group, and those who consumed the least to the PLANT group. If the analysis had been done based on the actual consumption of animal- and plant-sourced proteins, the differences between groups would have been clearer than the differences obtained with mismatched individuals affecting the group measures. However, it is also interesting that such mixing between the intervention groups occurred even though the instructions given to the participants regarding how to follow the diet were extremely detailed. This may indicate that participants had difficulties in interpreting or following the instructions. This supports the claim that large-scale dietary changes are difficult in practice [50, 51]. Changes in consumption patterns can be particularly difficult when it comes to high-protein foods because plant-based proteins are less digestible and may cause gastrointestinal symptoms. These potentially hindering factors deserve attention in research on dietary change. However, mixing between the intervention groups might also be a consequence of the individual variation in food consumption. For example, the participants were supposed to consume the delivered foods within a week, but the food diaries were only collected for four days. Thus, it might be that they ate either more animal or plant products during the diary days than the rest of the week, especially in the 50/50 group.

In this study, the climate impacts of diets were assessed as secondary outcomes of the nutritional intervention, in which the nutritional and health consequences of realised intervention diets were investigated. In previous work, we observed that some health benefits were achieved in both the 50/50 and PLANT groups, such as better lipid profile and higher fibre intake [33] and in the PLANT group lower concentrations of colorectal cancer risk markers, N-nitroso compounds [42]. However, some adverse health consequences such as lower vitamin B12 and iodine status and intake, as well as undesirable changes in bone turnover, were seen as a consequence of lower calcium and vitamin D intake, especially in the PLANT group [34, 35]. These changes in health indicators and nutrient intakes are a consequence of decreasing the amount dairy and meat dose-dependently in the diets. Calcium and vitamin D, usually from dairy products, are needed for proper bone function [52], whereas N-nitroso compounds are metabolites of red and processed meat [53].

We analysed correlations between climate impacts and health indicators that reflect biomarkers of risk for most important non-communicable diseases causing burden in the Western countries [39–41]. These indicators were risk of cardiovascular disease (blood lipids, blood pressure), type 2 diabetes (insulin, glucose), colorectal cancer (N-nitroso compounds), and osteoporosis (bone health; the ratio of markers for bone formation and resorption). Regarding the correlations, the results were mostly similar for the climate impact of diet as such and for energy-adjusted values (within an exception of total N-nitroso compounds where the correlation was not significant for the climate impact of diet as such). The dietary climate impact correlated positively with total and haem N-nitroso compounds and animal-sourced iron intake but negatively with fibre intake, indicating harmful consequences to gut health, particularly the risk of colorectal cancer. N-nitroso compounds and animal-sourced iron originate from red and processed meat, and especially beef is associated with a very high climate impact (per product mass) compared to other products. This supports the recommendation that the consumption of red and processed meat should be limited for both climate and health reasons (e.g [54]).

A significant correlation of climate impact with a positive status of bone turnover was observed, indicating that foods contributing to climate impact have beneficial effects on bone health. The climate impact and calcium intake had also a positive correlation. The intake of calcium is related to dairy products, which are widely consumed in Finland [36], particularly in liquid form. The climate impact of dairy products varies a lot (per product mass), with the climate impact of milk and other liquid dairy products being quite low, but those of cheese and butter being high (Supplement B). In the intervention, dairy product consumption was reduced in the 50/50 and PLANT diets along with meat. It is possible that the correlation between the climate impact of diet and calcium intake is also affected by meat consumption which is in turn related to the climate impact of the diet. This is supported by previous research, according to which a significant reduction in climate impact can be achieved with diets with little or no meat, but with relatively high milk consumption [48, 55, 56]. However, these analyses have not taken into account the interdependence between beef and milk production, on the basis of which the reduction of milk consumption to a moderate amount can be justified [55].

There was a positive correlation observed between climate impact and saturated fat. This reflects the fact that animal products, excluding low-fat dairy, are rich in saturated fat. Interestingly, however, many of the health indicators generally associated with high saturated fat intake, such as blood lipids and blood pressure [54], did not correlate with the climate impact (except the weak but significant correlation with non-HDL-cholesterol in an energy-adjusted analysis). The insignificant correlations with blood lipids or blood pressure may not be surprising, as not all protein sources with higher climate impact are necessarily high in saturated fat. A possible reason for not observing any significant correlations between climate impact and glucose metabolism markers, BMI, or blood pressure may be that our study subjects were generally healthy and not obese, as various factors in addition to dietary changes are reflected in these health indicators.

Based on these results, it is not obvious that the cross-sectionally assessed climate impact and health effects would go hand in hand. These results show that the climate impact of a diet can correlate with both positive or negative health biomarkers that are mediated through the eaten foods and their nutrients. To our knowledge, this has not been observed in previous studies.

This interpretation is also supported by a wider previous study of the nutrient composition of the same diets according to which the 50/50 group provided mostly an adequate amount of nutrients whereas in the PLANT diet the mean intakes did not meet the current Finnish reference values of some critical nutrients such as vitamin D and calcium [33–35, 37]. It is notable that the intake of iodine was fairly similar in 50/50 and PLANT groups but there were individuals below the reference values [35, 37]. Moreover, vitamin B12 intake exceeded the reference values in both groups, but there were individuals with inadequate intakes, especially in the PLANT group [35, 37]. Vitamin D is a common public health challenge, as the mean intakes were below the recommendations [34, 37] in all three study groups and 40% had inadequate vitamin D status [34, 52]. However, the intakes were lower in 50/50 and PLANT groups compared to the ANIMAL group [34]: if the amount of vitamin D fortified fluid milk in the diet is reduced, vitamin D should be received from other sources such as from fortified plant-based milk or supplements to ensure the adequate intake. The recommendations for vitamin B12 and calcium intake have increased in the latest Nordic Nutrition Recommendations 2023 [54]; if compared to these reference values, neither the 50/50 nor PLANT group reached the adequate intakes. Lower intakes of those critical nutrients in predominantly plant-based diets have also been observed previously [16, 57, 58]. Regarding the health outcomes, the PLANT diet provided both more health benefits such as a better lipid profile and lower concentrations of N-nitroso compounds [28]. On the other hand, the PLANT diet was also associated with some detrimental effects like accelerated bone turnover [29] which in the long run can increase fracture risk.

Even though these health impacts have also been observed in the previous studies with plant-based diets [6, 59–62], we cannot conclude that any of the diets is optimal from all three aspects: the environmental effects, dietary adequacy, and health effects. One also must take into account that the more we aim for sustainable diets by reducing the consumption of animal-sourced proteins, the more vulnerable our diets may become. This vulnerability arises from increased reliance on food fortification and dietary supplements to obtain critical nutrients, even though the overall health effects are likely to be positive. Overall, if a predominantly plant-based diet is to be followed, it is important to pay particular attention to the intakes of iodine, vitamins B12 and D, and calcium.

One strength of this study lay in its evaluation of realised instead of modelled diets. It was especially significant that the climate impacts and health biomarkers were studied from the same diets, so their relations could be analysed. However, a limitation of this study is that these were not self-selected diets, the evaluation of which would have yielded the most realistic results on the climate impact and health effects of a possible dietary transition to a more plant-based diet. Moreover, the analyses of health indicators are cross-sectional and relate to the climate impact, not the specific diets per se. Regarding health indicators, the markers were chosen based on their validity on the risk factor markers of several burdening non-communicable diseases. Besides well-established CVD and T2D risk markers, the markers of bone turnover describe acute or mid-term changes in bone metabolism, and they were chosen because in 12 weeks it is not possible to see changes in bone mineral density. Furthermore, the chosen markers were the ones recommended by the International Osteoporosis Foundation [63]. Similarly, N-nitroso compounds were used to estimate risk of colorectal cancer as they are carcinogenic compounds whose harmful effects have been established not only as molecular changes in CRC patients and laboratory animals but also in human cohort studies [53].

From a methodological perspective, one strength of this study was the use of different FUs in the assessment. In LCA, results are very sensitive with respect to the details of both the product system (diet composition) and the FU. The use of different FUs thus allows for a deeper understanding of the subject, which is why the use of several FUs is recommended [46]. In general, by using standardised energy content, e.g. 2,000 kcal or an amount of protein (e.g., one gram) as an FU, more accurate and comparable results can be obtained. These FUs also reflect at least some aspects of the nutritional quality of a diet, which is important in food LCA applications [46]. However, realised diets, such as the intervention diets in this study, differ qualitatively in many ways, which may be reflected in diet-specific nutrition and health biomarkers. Thus, the supposedly better accuracy of energy-adjusted or protein-based FUs can be misleading.

Finally, one limitation of this study is the scarcity of LCA results for a wide range of food products included in any realised diet. We used extensive data, but for many food products and dishes, we still had to use approximations. Furthermore, despite the additions we did to the source data, the final dataset was not fully harmonised. For example, we did not harmonise the methodological choices in the utilized literature such as emission models of agricultural production, impact assessment models, etc., because the data on imported products especially are expected to have large uncertainties due to the incompatibility of data sources. Thus, a harmonisation under these limitations would presumably not have resulted in increased accuracy. However, the choice of data was based on readily harmonised data sources as far as possible (Supplement B). For example, a database provided in [32 in Supplement B] was used, particularly for imported products. Data accuracy is always an issue in LCA studies, particularly in comparative product LCAs, but since this study focused on whole diets instead of single products, large uncertainties for individual products are unlikely to have outsized effects on the end result. However, this holds only under the assumption that the product grouping is sufficiently accurate and representative, taking into account the actual products sufficiently well [48, 49], as it is in this study. The shortcomings in data quality were therefore unlikely to be crucial.

## Conclusions

This study demonstrated that increasing the proportion of plant-sourced proteins in a diet leads to a reduction of the dietary climate impact, which is also supported by previous research. While such dietary changes were found to be generally safe, some concerning signals were detected even with moderate replacement of animal-sourced proteins with plant-sourced proteins. The relationship between climate impact and biomarkers for chronic diseases were ambiguous: climate impact had a positive correlation both with biomarkers for colorectal cancer, which is a harmful outcome, and a positive status of bone turnover, which is a beneficial outcome, and in addition, no correlation was observed for several other markers. Thus, further investigation on dietary climate impacts and health outcomes on larger populations is required.

## Electronic supplementary material

Below is the link to the electronic supplementary material.

Supplementary Material 1
