# Supplementary material for: Dietary climate impact correlates ambiguously with health biomarkers– a randomised controlled trial in healthy Finnish adults
Source: Eur J Nutr. 2025 Feb 18;64(2):95. doi: 10.1007/s00394-025-03609-w (PMC11836174; doi:10.1007/s00394-025-03609-w)
Supplement: Supplementary file 1 — Supplementary Material 1 [file 394_2025_3609_MOESM1_ESM.docx]

# Supplementary material to

# Dietary climate impact correlates ambiguously with health biomarkers – A randomised controlled trial in healthy Finnish adults

Merja Saarinen, Tiina Pellinen, Joel Kostensalo, Jouni Nousiainen, Katri Joensuu, Suvi T. Itkonen, Anne-Maria Pajari

# Supplement A

Additional information about randomized controlled trial (the ScenoProt intervention study).

**Table A1** Consumption frequencies of daily consumption of specific foods and food groups in the intervention diets based on the delivered food items and diet instructions^a^ [1].

|  | **ANIMAL** | **50/50** | **PLANT** |
| --- | --- | --- | --- |
| **Sources of animal proteins**  Main dishes containing minced meat (times/wk)  Main dishes containing whole meat (times/wk)  Main dishes containing sausage (times/wk)  Sausages and cold cuts, including processed poultry (g/d)  Pork and beef (g/d)  **Red and processed meat total (g/d)** | 2–3  2–3  1  35  64  **99** | 1–2  1–2  0–1  23  43  **65** | 0–1  0–1  0–1  11  21  **32** |
| Fish dishes (times/wk)  Fish (g/d) | 2  36 | 2  36 | 2  36 |
| Main dishes containing poultry (times/wk)  Poultry (g/d) | 2–3  43 | 1–2  29 | 1  14 |
| Eggs/wk (in dishes and pastries; boiled or fried)  Eggs (g/d) | 4  31 | 4  31 | 4  31 |
| Dairy products other than cheese (g/d) | 400 | 250 | 125 |
| Cheese (g/d) | 40 | 25 | 10–15 |
| **Sources of plant proteins**  Main dishes based on peas, lentils, chickpeas, tofu, crushed soya beans, or faba beans as main ingredients (times/wk) | 0–1 | 3–5 | 5–7 |
| Vegetable patties, pizza, mushroom dishes (portions/wk) | 1 | 2–3 | 2–3 |
| Nuts, almonds, and seeds (g/d) | occasionally | 16 | 34 |
| Plant-based dairy-like products (other than cheese), g/d | 0 | 150 | 250 |
| Bread (rye and oat/wheat bread; slices of bread/d)  Bread (g/d) | 4–5  120–150 | 6  180 | 7  210 |
| Porridge and muesli (g/d, dry weight) | 40 | 40–60 | 40–80 |
| Whole-grain rice, pasta, couscous, quinoa (g/d, dry weight) | 70 | 70–105 | 70–140 |
| Potatoes (g/d, cooked) | 120 | 0–120 | 0–120 |

^a^Average daily consumption is presented as g/d. ANIMAL, a diet containing 70% animal and 30% plant source proteins; 50/50, a diet containing equal proportions (50:50) of animal and plant-based protein sources; PLANT, a diet containing 30% animal and 70% plant proteins.

## Reference

1 Pellinen T, Päivärinta E, Isotalo J, Lehtovirta M, Itkonen ST, Korkalo L, Erkkola M, Pajari AM (2022) Replacing Dietary Animal-Source Proteins with Plant-Source Proteins Changes Dietary Intake and Status of Vitamins and Minerals in Healthy Adults: A 12-Week Randomized Controlled Trial. European Journal of Nutrition 61 (3):1391–1404. <https://doi.org/10.1007/s00394-021-02729-3>

# Supplement B

## Climate impact coefficients for foods

The climate impact of food products and meals included in the intervention diets, a total of 3,075 products classified in 107 product groups, was estimated based on the literature data. The greenhouse gas emissions caused by the primary production, processing, packaging, transport, and retail stages of the production chain were included. To account for the variation in products within product categories and the existing literature sources, minimum and maximum values were listed separately for each product category (Table B1).

For fresh fruit, the climate impact was calculated for the edible part of the products by multiplying the emission factors by fruit-specific shares of the edible portions obtained from [1]. For mixed vegetable salads, the average of the emission factors of fresh vegetable groups (lettuce, spinach, kale, Chinese cabbage, white cabbage, broccoli, tomato, cucumber, onion, garlic, carrot, beetroot, pumpkin, asparagus, fennel and celery, eggplant, bell pepper, and artichoke) was used. Similarly, for smoothies containing vegetables, fruit, and berries, the average of fresh vegetable groups and fruit and berries was calculated. For the average of emission factors of fruit, the fresh fruit groups included were citrus fruit, banana, apple, pear, peach, plum, apricot, cherry, pineapple, grapes, melon, avocado, kiwi fruit, date, coconut, fig, mango, and papaya. The average of berries was calculated as the average of the available literature values for Finnish (strawberries, blueberries, and blackcurrant) and foreign berries (strawberries, blueberries, raspberries, cranberries, and currants). For smoothies containing additionally plant-based drinks or yoghurt, the average of the emission factors of smoothies containing vegetables, fruit and berries, and plant-based drinks or yoghurt respectively was used. For the primary production of cooked root vegetable side dishes, the mean of carrots and turnips, other root vegetables, and onions was calculated.

For dried fruit and berries, the average of fruit and berries was multiplied by four [2]. For the primary production of canned fruit and fruit salads, the average of fruit was used. The ‘savoury confectionery and hamburgers’ group was divided into products containing red meat, products containing poultry meat, and vegetarian products. The average values for corresponding ready-made meal groups were used for them: cooked industrial meat dishes; cooked industrial broiler and turkey dishes; and other cooked industrial vegetable dishes.

For some products, separate global warming potential (GWP) coefficients were used for industrial processing to include the climate impact of this phase the life cycle of a product, because no literature source that covered both the primary production and processing stages was found. Such products were roasted nuts, canned vegetables and fruit, and meat steaks and chops – see comments in Table B1. Additionally, for some products, separate GWP coefficients were used for transport and packaging, because no literature source that covered this and the previous stages of the production chain (primary production and processing) was found. Such products were the abovementioned product groups, as well as eggs and egg dishes – see comments in Table B1. The climate impact of the retail stage of the production chain was missing from many of the literature sources. A list of the product groups and literature sources that were used to estimate the climate impact of the retail stage is presented in Table B2.

The climate impact of home cooking and cool storage was calculated using the average electricity consumption values of electrical appliances and preparation times [3] and the climate impact of Finnish average electricity [4].

**Table B1.** The minimum and maximum values of total climate impacts (kg CO_2_-eq./kg) of food product categories.

| Product category | Number of products in product category | Total GHG | | References | | Number of used data sources | Comment |
| --- | --- | --- | --- | --- | --- | --- | --- |
|  |  | Min | Max | Min | Max |  |  |
| Nuts | 21 | 0.8 | 5.5 | [5] | [6] | 8 | a |
| Seeds | 12 | 0.9 | 2.5 | [7] | [5] | 3 | b |
| Soy based milk, yoghurt | 26 | 0.4 | 1.7 | [8] | [9] | 5 |  |
| Oat based milk, yoghurt | 21 | 0.4 | 1.3 | [10] | [11] | 3 |  |
| Nut based drinks | 35 | 0.7 | 0.8 | [12] | [13] | 3 |  |
| Other plant-based drinks | 4 | 0.6 | 0.6 | [14] | [14] | 1 | c |
| Fresh vegetables and salads | 89 | 0.1 | 4.5 | [15] | [16] | 26 | d |
| Smoothies containing vegetables, fruit, and berries | 5 | 0.1 | 4.5 | [17] | [16] | 44 |  |
| Vegetable main dishes | 52 | 0.3 | 2.7 | [18] | [18] | 3 |  |
| Legume side dishes | 8 | 0.8 | 1.6 | [19] | [5] | 4 |  |
| Vegetable side dishes | 88 | 0.1 | 4.5 | [15] | [16] | 44 | d |
| Pea and bean dishes | 93 | 0.7 | 2.5 | [20] | [21] | 5 |  |
| Soybean dishes | 27 | 1.0 | 1.4 | [20] | [20] | 1 |  |
| Mushrooms and mushroom dishes | 24 | 0.1 | 4.2 | [2] | [22] | 5 |  |
| Pulled oats dishes | 21 | 0.7 | 2.5 | [20] | [21] | 5 | e |
| Mayonnaise-based and main course salads | 84 | 0.7 | 3.7 | [11] | [5] | 4 | f |
| Canned vegetables | 10 | 0.7 | 3.7 | [11] | [5] | 4 |  |
| Potato, boiled and mashed | 31 | 0.4 | 0.5 | [11] | [23] | 2 |  |
| Potatoes, fried and potato dishes | 27 | 0.4 | 4.5 | [11] | [24] | 5 |  |
| Fresh fruit | 30 | 0.1 | 3.0 | [25] | [5] | 17 |  |
| Berries | 13 | 0.0 | 1.6 | [17] | [26] | 12 |  |
| Berry and fruit soups | 47 | 0.5 | 1.4 | [27] | [23] | 7 | g |
| Berry and fruit pies | 24 | 1.3 | 1.9 | [28] | [29] | 3 | h |
| Canned fruit and fruit salads | 26 | 0.3 | 3.2 | [25] | [5] | 17 | i |
| Jams, marmalades | 37 | 0.3 | 3.2 | [25] | [5] | 25 | i |
| Fruit and berry juices | 9 | 0.5 | 14 | [27] | [23] | 7 |  |
| Berry and fruit smoothies | 16 | 0.1 | 4.5 | [17] | [16] | 44 | j |
| Breakfast cereals, sweetened with sugar | 24 | 0.8 | 3.1 | [30] | [31] | 4 |  |
| Porridge | 111 | 0.2 | 0.3 | [11] | [11] | 1 |  |
| Pasta side dish | 35 | 0.5 | 0.5 | [11] | [11] | 1 |  |
| Rice and other cereal side dishes | 28 | 2.9 | 3.9 | [11] | [11] | 1 |  |
| Pizza | 40 | 1.7 | 4.7 | [32] | [33] | 3 |  |
| Biscuits | 43 | 0.9 | 4.5 | [29] | [34] | 3 |  |
| Cakes, pastries | 54 | 1.3 | 1.9 | [28] | [29] | 3 | h |
| Savoury confectionery, hamburgers | 65 | 1.0 | 27.4 | [35] | [36] | 10 |  |
| Pancakes, crepes | 28 | 1.2 | 6.2 | [37] | [38] | 6 | k |
| Breakfast cereals, unsweetened | 20 | 0.8 | 1.8 | [30] | [11] | 3 |  |
| Margarine >=60% | 23 | 1.4 | 1.9 | [39] | [39] | 1 |  |
| Margarine <60% | 13 | 1.4 | 1.9 | [39] | [39] | 1 |  |
| Mixed fats <60% | 3 | 7.2 | 7.2 | [40] | [40] | 1 |  |
| Butter | 1 | 2.3 | 10.9 | [41] | [40] | 4 |  |
| Mixed fats >=60% | 15 | 7.2 | 7.2 | [40] | [40] | 1 |  |
| Salad dressings, plant oils, mayonnaise, other fats | 41 | 1.1 | 6.3 | [42] | [41] | 5 |  |
| Other fats | 6 | 1.1 | 6.3 | [42] | [41] | 5 | l |
| Other spreads | 17 | 0.5 | 2.6 | [38] | [21] | 3 | m |
| Fish, fish fillets | 61 | 1.3 | 5.2 | [18] | [11] | 3 |  |
| Fish casseroles | 10 | 1.3 | 2.4 | [18] | [11] | 2 |  |
| Fish soups | 17 | 1.3 | 2.4 | [18] | [11] | 2 | n |
| Other cooked fish dishes and products | 25 | 1.4 | 2.5 | [18] | [11] | 5 |  |
| Dry-cured and cold smoked fish | 4 | 5.1 | 5.1 | [18] | [11] | 1 |  |
| Cooked industrial fish products | 31 | 1.4 | 8.1 | [18] | [11] | 7 |  |
| Cold cuts | 22 | 0.5 | 26.2 | [36] | [36] | 6 | k |
| Mince meat dishes | 65 | 3.0 | 12.0 | [18] | [36] | 4 |  |
| Broiler and turkey dishes | 40 | 0.8 | 4.9 | [35] | [18] | 4 |  |
| Meat soups | 31 | 3.1 | 3.1 | [18] | [18] | 1 |  |
| Meat casseroles | 32 | 2.8 | 7.0 | [20] | [20] | 1 |  |
| Meat stews and sauces | 74 | 3.0 | 3.0 | [18] | [18] | 1 |  |
| Sausage dishes | 8 | 2.2 | 17.2 | [36] | [36] | 5 |  |
| Steaks and chops | 50 | 5.0 | 86.0 | [18] | [43] | 14 | o |
| Offal and blood dishes | 8 | 0.5 | 2.4 | [36] | [20] | 2 |  |
| Other broiler and turkey dishes | 75 | 0.8 | 4.9 | [35] | [18] | 4 |  |
| Milk, fat-free | 12 | 1.2 | 2.5 | [11] | [41] | 7 |  |
| Milk, 0.1–2% fat | 15 | 1.2 | 2.5 | [11] | [41] | 7 |  |
| Milk, >2% fat | 6 | 1.2 | 2.5 | [11] | [41] | 7 |  |
| Cream | 10 | 3.3 | 5.5 | [11] | [40] | 2 |  |
| Butter milk, fat-free | 4 | 0.3 | 2.0 | [44] | [45] | 5 |  |
| Butter milk, 0.1–2.5% fat | 11 | 1.3 | 2.0 | [44] | [45] | 5 |  |
| Quark | 41 | 1.3 | 2.0 | [44] | [45] | 5 |  |
| Yoghurt | 50 | 1.3 | 2.0 | [44] | [45] | 5 |  |
| Curdled milk | 7 | 1.3 | 2.0 | [44] | [45] | 5 |  |
| Cheese, hard | 28 | 6.3 | 16.8 | [46] | [41] | 5 |  |
| Cream cheese | 19 | 3.0 | 7.0 | [46] | [40] | 4 |  |
| Soft cheese | 4 | 3.0 | 7.0 | [46] | [40] | 4 |  |
| Ice cream and milk desserts | 41 | 2.3 | 3.8 | [47] | [48] | 6 |  |
| Milk-based sauces | 40 | 1.3 | 2.0 | [44] | [45] | 5 | p |
| Yoghurt and milk-based smoothies | 42 | 0.2 | 4.6 | [17] | [16] | 49 |  |
| Sugar and honey | 6 | 0.5 | 1.5 | [23] | [41] | 5 |  |
| Sweets | 17 | 2.4 | 3.9 | [49] | [49] | 1 |  |
| Chocolate | 17 | 1.8 | 4.4 | [50] | [41] | 4 |  |
| Sweets, sweetened with xylitol | 8 | 2.4 | 3.9 | [49] | [49] | 1 | q |
| Dried fruit and berries, snacks | 32 | 0.1 | 11.9 | [25] | [5] | 29 | r |
| Dietary supplements, natural products | 40 | - | - | - | - | - |  |
| Spices, piquant sauces, dietary products, meal replacements | 57 | 0.2 | 8.9 | [51] | [40] | 3 |  |
| Animal-based protein supplements and protein bars | 8 | 6.2 | 8,9 | [40] | [40] | 1 | s |
| Plant-based protein supplements and protein bars | 1 | 6.2 | 8.9 | [40] | [40] | 1 | s |
| Vegetable soups and sauces | 55 | 0.7 | 1.0 | [52] | [52] | 1 | t |
| Bottled water | 6 | 0.2 | 0.5 | [53] | [54] | 2 |  |
| Coffee, tea and other hot drinks | 23 | 0.0 | 3.0 | [55] | [56] | 11 |  |
| Fruit and berry juices | 16 | 0.5 | 1.4 | [27] | [23] | 7 |  |
| Juices, sweetened with artificial sweeteners | 4 | 0.5 | 1.4 | [27] | [23] | 7 | g |
| Soft drinks | 7 | 0.3 | 1.4 | [54] | [49] | 4 |  |
| Soft drinks, sweetened with artificial sweeteners | 3 | 0.3 | 1.4 | [54] | [49] | 4 |  |
| Soft drinks, cola | 1 | 0.3 | 1.4 | [57] | [49] | 2 |  |
| Soft drinks, cola, sweetened with artificial sweeteners | 1 | 0.3 | 1.4 | [57] | [49] | 2 |  |
| Alcoholic drinks | 22 | 0.5 | 4.0 | [58] | [59] | 13 |  |
| Alcohol-free cider and beer | 1 | 0.5 | 0.9 | [58] | [58] | 5 |  |
| Eggs and egg dishes | 53 | 1.3 | 5.0 | [60] | [61] | 12 |  |
| Boiled eggs | 3 | 1.3 | 5.0 | [60] | [61] | 12 |  |
| Rye bread | 33 | 1.1 | 1.7 | [11] | [62] | 2 |  |
| Mixed-grain bread | 116 | 0.7 | 1.3 | [63] | [11] | 3 |  |
| Wheat bread | 28 | 0.5 | 1.3 | [44] | [41] | 4 |  |
| Buns, doughnuts | 40 | 1.3 | 1.9 | [28] | [29] | 3 |  |
| Cooked industrial meat dishes | 43 | 2.9 | 27.4 | [64] | [36] | 7 |  |
| Cooked industrial broiler and turkey dishes | 10 | 1.0 | 5.6 | [35] | [37] | 5 |  |
| Cooked industrial legume dishes | 22 | 0.5 | 2.6 | [38] | [21] | 3 |  |
| Cooked industrial soybean dishes | 14 | 1.0 | 2.7 | [65] | [21] | 6 |  |
| Cooked industrial vegetable dishes | 58 | 1.2 | 6.2 | [37] | [38] | 6 |  |

a For industrial processing of roasted nuts, the climate impact was estimated according to [11]. For packaging and transport, the climate impact was estimated according to [38] and [41]

b For packaging and transport, the climate impact was estimated according to [38] and [41]

c The values are based on coconut milk.

d For packaging and transport, the climate impact was estimated based on leaf vegetables [23] cabbages [11], other vegetables [41]

e The same values were used as for pea and bean dishes

f The same values were used as for canned vegetables

g The same values were used as for fruit and berry juices

h The same values were used as for buns and doughnuts

i For primary production, the same values were used as for fruit. For processing, the climate impact was estimated based on [66] and [67]

j The same values were used as for smoothies containing vegetables, fruit, and berries

k The same values were used as for industrial vegetable dishes

l The same values were used as for salad dressings and plant oils

m The same values were used as for industrial legume dishes

n The same values were used as for fish casseroles

o For processing, packaging, and transport, the climate impact was estimated based on [41]

p The same values were used as for yoghurt

q The same values were used as for sweets

r Calculated as the average of fruit and berries multiplied by [8], according to [21]

s The same values were used as for milk powder

t The same values were used as for ketchup

**Table B2.** Literature sources used for estimating the climate impact caused by energy consumption during the retail stage of the production chain.

| **Product group** | Reference | Comment |
| --- | --- | --- |
| Nuts and seeds | [41] |  |
| Plant-based drinks | [38, 41] |  |
| Leafy vegetables | [23, 68] |  |
| Other vegetables | [41] |  |
| Bean side dishes | [23] |  |
| Canned vegetables and fruit | [38] |  |
| Fresh fruit | [41, 69] |  |
| Fruit and berry juices | [70] |  |
| Breakfast cereals, porridge, pasta side dish | [41] | a |
| Rice | [41] |  |
| Biscuits | [41] | a |
| Cakes, pastries | [41] | b |
| Savoury confectionery, hamburgers | [33, 38, 41] |  |
| Margarine, butter, mixed fats | [41] | c |
| Salad dressings, plant oils, mayonnaise, other fats | [41] | d |
| Cooked fish dishes and products, dry-cured and cold smoked fish | [41] | e |
| Cold cuts, steaks and chops, sausages | [41] | f |
| Milk, cheese, other dairy products | [41] |  |
| Ice cream (milk and plant-based) | [37, 48, 71, 72, 47] |  |
| Sugar, honey, sweets, chocolate | [41] |  |
| Dried fruit and berries, chips and snacks, spices and piquant sauces, milk powder | [41] | a |
| Soft drinks and bottled water | [54] |  |
| Beer | [54, 73, 58, 74, 41] |  |
| Wine and spirits | [41] |  |
| Eggs and egg dishes | [41] |  |
| Bread and other bakery products, rice, breakfast cereals | [41] |  |
| Other bakery products | [41] | b |
| Cooked industrial meat dishes | [33] |  |
| Cooked industrial broiler and turkey dishes | [41] | g |
| Cooked industrial legume dishes | [38] |  |
| Cooked industrial soybean dishes | [38, 41] |  |
| Cooked industrial vegetable dishes | [38] |  |

a As oat flakes

b As bread

c As butter

d As plant oils

e As raw fish

f As raw pork

g As raw poultry meat

## References

1 Sääksjärvi K, Reinivuo, H (2004) Ruokamittoja. (In Finnish) (Food Measures) (Publications of National Institute for Health and Welfare) B15/2004. Kansanterveyslaitoksen julkaisuja, Helsinki.

2 Hartikainen H, Pulkkinen H (2016) Summary of the chosen methodologies and practices to produce GHGE-estimates for an average European diet. Natural resources and bioeconomy studies 58/2016.

3 Adato Energia Oy (2013) Kotitalouksien sähkönkäyttö 2011 (Energy consumption of households 2011). Available at: <https://www.vattenfall.fi/energianeuvonta/sahkonkulutus/sahkolaitteiden-energiankulutus/>

4 Yrjänäinen H (2011) Sähkön hiilijalanjälki Suomessa (In Finnish) (Carbon footprint of electricity in Finland). MSc thesis, Tampere University of technology. Tampere, Finland. 76 pp.

5 Audsley E, Brander M, Chatterton J, Murphy-Bokern D, Webster C, Williams A (2009) How low can we go? An assessment of greenhouse gas emissions from the UK food system and the scope to reduce them by 2050. WWF-UK.

6 de Figueirêdo MCB, Potting J, Serrano LAL, Bezerra MA, da Silva Barros V, Gondim RS, Nemecek T (2016) Environmental assessment of tropical perennial crops: the case of the Brazilian cashew. Journal of cleaner production, 112:131–140. <https://doi.org/10.1016/j.jclepro.2015.05.134>

7 Figueiredo F, Castanheira ÉG, reire F (2017) Life-cycle assessment of irrigated and rainfed sunflower addressing uncertainty and land use change scenarios. Journal of Cleaner Production, 140:436–444. <https://doi.org/10.1016/j.jclepro.2016.06.151>

8 Wernet G, Bauer C, Steubing B, Reinhard J, Moreno-Ruiz E, Weidema B (2016) The ecoinvent database version 3 (part I): overview and methodology. The International Journal of Life Cycle Assessment 21:1218–1230. <https://doi.org/10.1007/s11367-016-1087-8>

9 Silvenius F, Katajajuuri JM, Koivupuro HK, Nurmi P, Virtanen Y, Grönman K, Soukka R (2011) Elintarvikkeiden pakkausvaihtoehtojen ympäristövaikutukset. FutupackEKO2010-hanke.

10 Oatly (2018) <https://www.oatly.com/fi/klimatavtryck/how-and-why> and <https://www.oatly.com/fi/products/ikaffe>

11 Saarinen M, Sinkko T, Joensuu K, Silvenius F, Ratilainen A (2014) Ravitsemus ja maaperävaikutukset ruoan elinkaariarvioinnissa: SustFoodChoice-hankkeen loppuraportti. (In Finnish) (Nutrition and soil impacts in life cycle assessment of food: final report of the SustFoodChoice project). MTT report 186. <http://urn.fi/URN:ISBN:978-952-487-540-0>

12 Winans KS, Macadam-Somer I, Kendall A, Geyer R, Marvinney E (2020) Life cycle assessment of California unsweetened almond milk. The International Journal of Life Cycle Assessment, 25**,**577–587. <https://doi.org/10.1007/s11367-019-01716-5>

13 Henderson A, Unnasch S (2017) Life cycle assessment of ripple non-dairy Milk. Life cycle associates LCA, 6121, 2017. <https://www.ripplefoods.com/pdf/Ripple_LCA_Report.pdf>

14 Clune S, Crossin E, Verghese K (2017) Systematic review of greenhouse gas emissions for different fresh food categories. Journal of Cleaner Production, 140:766–783.  <https://doi.org/10.1016/j.jclepro.2016.04.082>

15 Romero-Gámez M, Audsley E, Suárez-Rey EM (2014) Life cycle assessment of cultivating lettuce and escarole in Spain. Journal of cleaner production, 73:193–203. <https://doi.org/10.1016/j.jclepro.2013.10.053>

16 Stoessel F, Juraske R, Pfister S, Hellweg S (2012) Life cycle inventory and carbon and water foodprint of fruits and vegetables: application to a Swiss retailer. Environmental science & technology, 46(6), 3253-3264.

17 Girgenti V, Peano C, Baudino C, Tecco N (2014) From “farm to fork” strawberry system: Current realities and potential innovative scenarios from life cycle assessment of non-renewable energy use and green house gas emissions. Science of the Total Environment, 473, 48-53.  <https://doi.org/10.1016/j.scitotenv.2013.11.133>

18 Pulkkinen H, Roininen T, Katajajuuri, JM, Järvinen M (2014) Development of Climate Choice Lunch concept for restaurants based on carbon footprinting. In Proceedings of the 9th International Conference on Life Cycle Assessment in the Agri-Food sector.

19 Milà i Canals L, Muñoz I, Hospido A, Plassmann K, McLaren S (2008) Life Cycle Assessment (LCA) of Domestic vs. Imported Vegetables. Casestudies on broccoli, salad crops and green beans. (Working paper) Available at: <http://www.surrey.ac.uk/ces/files/pdf/0108_CES_WP_RELU_Integ_LCA_local_vs_global_vegs.pdf>

20 Saarinen M, Kurppa S, Virtanen Y, Usva K, Mäkelä J, Nissinen A, (2012) Life cycle assessment approach to the impact of homemade, ready-to-eat and school lunches on climate and eutrophication. J. Clean. Prod. 28, 177e186. <https://doi.org/10.1016/j.jclepro.2011.11.038>

21 Head M, Sevenster M, Croezen H (2011) Life cycle impacts of proteinrich foods for superwijzer. Delft

22 Leiva FJ, Saenz-Díez, JC, Martínez E, Jiménez E, Blanco J (2014) Environmental impact of Agaricus bisporus cultivation process. European Journal of Agronomy. Elsevier Ltd. 71: 141-148. ISSN: 1161-0301. <https://doi.org/10.1016/j.eja.2015.09.013>

23 Räsänen K, Saarinen M, Kurppa S, Silvenius F, Riipi I, Nousiainen R, Erälinna L, Mattinen L, Jaakkola S, Lento S, Mäkinen-Hankamäki S (2014) Lähiruuan ekologisten vaikutusten selvitys. (In Finnish)( Study of the ecological effects of local food) MTT Report 145, Jokioinen. <http://urn.fi/URN:ISBN:978-952-487-538-7>

24 Usva K, Saarinen M, Katajajuuri, JM, Kurppa S (2009) Supply chain integrated LCA approach to assess environmental impacts of food production in Finland. Agricultural and Food Science, 18(3-4), 460-476.

25 González AD, Frostell, B, Carlsson-Kanyama A (2011) Protein efficiency per unit energy and per unit greenhouse gas emissions: potential contribution of diet choices to climate change mitigation. Food policy, 36 (5):562–570. <https://doi.org/10.1016/j.foodpol.2011.07.003>

26 Soode E, Lampert P, Weber-Blaschke G, Richter K (2015) Carbon footprints of the horticultural products strawberries, asparagus, roses and orchids in Germany. Journal of cleaner production:87,:168–179. <https://doi.org/10.1016/j.jclepro.2014.09.035>

27 Knudsen MT, Almeida GF, Langer V, Santiago de Abreu L, Hallberg N (2011) Environmental assessment of organic juice imported to Denmark: a case study on oranges (Citrus sinensis) from Brazil. Organic Agriculture – Official journal of The International Society of Organic Agriculture Research, 1 (3):167–185.  <https://doi.org/10.1007/s13165-011-0014-3>

28 Barilla (2012) Dichiarazione Ambientale di Prodotto applicata ai Pavesini. Available at: <http://gryphon.environdec.com/data/files/6/8527/epd234it.pdf>

29 Barilla (2013) Chicchi di Cioccolato. Dichiarazione Ambientale di Prodotto. Available at: <http://gryphon.environdec.com/data/files/6/9997/epd419_Mulino_Bianco_Chicchi%20di%20cioccolato.pdf>

30 Raisio (2019) <https://www.elovena.fi/vastuullisuus>

31 Jeswani HK, Burkinshaw R, Azapagic A (2015) Environmental sustainability issues in the food–energy–water nexus: Breakfast cereals and snacks. Sustainable Production and Consumption, 2:17–28.

32 Masset G, Soler LG, Vieux F, Darmon N (2014) Identifying sustainable foods: the relationship between environmental impact, nutritional quality, and prices of foods representative of the French diet. Journal of the Academy of Nutrition and Dietetics, 114 (6):862–869. <https://doi.org/10.1016/j.jand.2014.02.002>

33 Berners-Lee M, Hoolohan C, Cammack H, Hewitt CN (2012) The relative greenhouse gas impacts of realistic dietary choices. Energy policy 43:184–190. <https://doi.org/10.1016/j.enpol.2011.12.054>

34 Noya LI, Vasilaki V, Stojceska V, Gonzalez-García S, Kleynhans C, Tassou S, Moreira MT, Katsou E (2018) An environmental evaluation of food supply chain using life cycle assessment: A case study on gluten free biscuit products. Journal of cleaner production, 170:451–461. <https://doi.org/10.1016/j.jclepro.2017.08.226>

35 Davis J, Sonesson U (2008) Life cycle assessment of integrated food chains—a Swedish case study of two chicken meals. The International Journal of Life Cycle Assessment, 13:574–584. <https://doi.org/10.1007/s11367-008-0031-y>

36 Scholz K (2013) Carbon footprint of retail food wastage. A case study of six Swedish retail stores. Independent thesis 2013:05. SLU, Sveriges lantbruksuniversitet, Uppsala, Sweden.

37 Berlin J, Sund V (2010) Environmental life cycle assessment (LCA) of ready meals: LCA of two meals; pork and chicken & screening assessments of six ready meals. SIK Institutet för livsmedel och bioteknik. SIK-Report No. 804 2011.

38 Blonk H, Kool A, Luske B, de Waart S (2008) Environmental effects of protein-rich food products in the Netherlands – Consequences of animal protein substitutes. Blonk consultants.

39 Nilsson K, Flysjö A, Davis J, Sim S, Unger N, Bell S (2010) Comparative life cycle assessment of margarine and butter consumed in the UK, Germany and France. The International Journal of Life Cycle Assessment, 15 (9):916–926. <https://doi.org/10.1007/s11367-010-0220-3>

40 Flysjö A (2012) Greenhouse gas emissions in milk and dairy product chains improving the carbon footprint of dairy products. PhD thesis. Science and Technology. Available at: <http://pure.au.dk/portal/files/45485022/Anna_20Flusj_.pdf>

41 Poore J, Nemecek T (2018) Reducing food’s environmental impacts through producers and consumers. Science, 360 (6392):987–992.

42 Tsarouhas P, Achillas C, Aidonis D, Folinas D, Maslis V (2015) Life Cycle Assessment of olive oil production in Greece. Journal of cleaner production, 93:75–83. <https://doi.org/10.1016/j.jclepro.2015.01.042>

43 Ripoll-Bosch R, De Boer IJM, Bernués A, Vellinga TV (2013) Accounting for multi-functionality of sheep farming in the carbon footprint of lamb: a comparison of three contrasting Mediterranean systems. Agricultural Systems, 116:60–68. <https://doi.org/10.1016/j.agsy.2012.11.002>

44 Lindenthal T, Markut T, Hörtenhuber S, Theurl M, Rudolph G (2010) Greenhouse gas emissions of organic and conventional foodstuffs in Austria, 2010. Available at: <https://www.fibl.org/fileadmin/documents/de/oesterreich/arbeitsschwerpunkte/Klima/lca_confernce_abstract_lindenthal_1003.pdf>

45 González-García S, Castanheira EG, Dias AC, Arroja L (2013) Environmental life cycle assessment of a dairy product: the yoghurt. Int J LCA 18 (4):796–811. <https://doi.org/10.1007/s11367-012-0522-8>

46 EDA (European Dairy Association) (2018) Product Environmental Footprint Category Rules for dairy products.

47 Zheng W (2010) The carbon footprint of ice cream and its mitigating options for Unilever in China

48 Konstantas A, Stamford L, Azapagic A (2019) Environmental impacts of ice cream. Journal of Cleaner Production, 209:259–272. <https://doi.org/10.1016/J.JCLEPRO.2018.10.237>

49 Nilsson K, Sund V, Florén B (2011) The environmental impact of the consumption of sweets, crisps and soft drinks. TemaNord 2011:509. SBN 978-92-893-2197-6

50 Jungbluth N, König A (2014) Environmental impacts of chocolate in a life cycle perspective. ESU-services Ltd.

51 Climatop (2009) Klimabilanz: Speisesalz im Kanton Waadt. Switzerland.

52 Andersson K, Ohlsson T (1999) Including environmental aspects in production development: a case study of tomato ketchup. LWT-Food Science and Technology, 32 (3):134–141. <https://doi.org/10.1006/fstl.1998.0513>

53 Finnspring (2010) Vastuullisuus. (In Finnish) (Responsibility) <https://finnspring.fi/yritys/vastuullisuus.html>

54 Amienyo D (2012) Life Cycle Sustainability Assessment in the UK Beverage Sector. A thesis submitted to the University of Manchester for the degree of Doctor of Philosophy in the School of Chemical Engineering and Analytical Science 2012, School of Chemical Engineering and Analytical Science.

55 Azapagic A, Bore J, Cheserek B, Kamunya S, Elbehri A (2016) The global warming potential of production and consumption of Kenyan tea. Journal of Cleaner Production, 112:4031–4040.  <https://doi.org/10.1016/j.jclepro.2015.07.029>

56 Büsser S, Jungbluth N (2009) The role of flexible packaging in the life cycle of coffee and butter. Int J LCA. 14 (sup 1):S80-S91. <https://doi.org/10.1007/s11367-008-0056-2>

57 Sinclair RJ (2009) **Greenhouse gas footprinting and berryfruit production: Business Case.** Landcare Contract Report LC64 prepared for the Ministry of Agriculture and Forestry Sustainable Farming Fund and Blackcurrants NZ Ltd, and the New Zealand Boysenberry Council, Wellington, New Zealand.

58 BIER (2012) <http://www.bieroundtable.com/wp-content/uploads/49d7a0_70726e8dc94c456caf8a10771fc31625.pdf>

59 Vázquez-Rowe I, Villanueva-Rey P, Moreira MT, Feijoo G (2012) Environmental analysis of Ribeiro wine from a timeline perspective: Harvest year matters when reporting environmental impacts. Journal of Environmental Management 98:73–83. <https://doi.org/10.1016/j.jenvman.2011.12.009>

60 Wiedemann SG, McGahan E J (2011) Environmental assessment of an egg production supply chain using life cycle assessment. Australian Egg Corporation Limited: Sydney.

61 Williams AG, Audsley E, Sanders DL (2006) Determining the environmental burdens and resource use in the production of agricultural and horticultural commodities, Main Report, Defra Research project IS0205, Bedford: Cranfield University and Defra. Available at: [www.silsoe.cranfield.ac.uk](http://www.silsoe.cranfield.ac.uk)

62 Silvenius F, Grönroos J, Kankainen M, Kurppa S, Mäkinen T, Vielma, J (2017) Impact of feed raw material to climate and eutrophication impacts of Finnish rainbow trout farming and comparisons on climate impact and eutrophication between farmed and wild fish. Journal of cleaner production, 164:1467–1473. <https://doi.org/10.1016/j.jclepro.2017.07.069>

63 Espinoza-Orias N, Stichnothe H, Azapagic A (2011) The carbon footprint of bread. The International Journal of Life Cycle Assessment, 16 (4):351–365. https://doi.org/10.1007/s11367-011-0271-0

64 Davis J, Sonesson U, Baumgartner DU, Nemecek T (2010) Environmental impact of four meals with different protein sources: Case studies in Spain and Sweden. Food Research International, 43:1874–1884. <https://doi.org/10.1016/j.foodres.2009.08.017>

65 Jalotofu (2014) <https://jalotofu.fi/jalofoods/vastuullisuus/>

66 Biel A, Bergström K, Carlsson-Kanyama A, Fuentes C, Grankvist G, Lagerberg FC, Fogelberg C, Shanahan H, Solér, C (2006) Environmental information in the food supply system. ISRN FOI. Available at: <http://www.fcrn.org.uk/sites/default/files/Environmental_information_in_the_food_supply_system.pdf>

67 Del Borghi A, Gallo M, Strazza C, Del Borghi M (2014) An evaluation of environmental sustainability in the food industry through Life Cycle Assessment: the case study of tomato products supply chain. Journal of Cleaner Production, 78:121–130. <https://doi.org/10.1016/j.jclepro.2014.04.083>

68 Silvenius F, Usva K, Katajajuuri JM, Jaakkonen AK (2019) Kasvihuonetuotteiden ilmastovaikutuslaskenta 2004 ja 2017 todellisten energiankulutustilastojen perusteella sekä vesijalanjälki. (In Finnish) (Climate impact calculation of greenhouse products in 2004 and 2017 based on actual energy consumption statistics and water footprint.) Commissioned study for the Finnish Gardeners' Association. Available at: <https://kauppapuutarhaliitto.fi/wp-content/uploads/2019/05/Kasvihuonetuotannon-ilmastovaikutus-tutkimus2019.pdf>

69 Vinyes E, Asin L, Alegre S, Muñoz P, Boschmonart J, Gasol CM (2017) Life Cycle Assessment of apple and peach production, distribution and consumption in Mediterranean fruit sector. Journal of cleaner production, 149:313–320. <https://doi.org/10.1016/j.jclepro.2017.02.102>

70 Dwivedi P, Spreen T, Goodrich-Schneider R (2012) Global warming impact of Florida’s Not-From-Concentrate (NFC) orange juice. Agric Syst 108:104–111. <https://doi.org/10.1016/j.agsy.2012.01.006>

71 Sheane R, Lewis K, Hall P, Holmes-Ling P, Kerr A, Stewart K, Webb D (2011) Identifying opportunities to reduce the carbon footprint associated with the Scottish dairy supply chain – Main report. Edinburgh: Scottish Government.

72 Wieriks P, Vosbeek M, Ginsel R, Hofman Y (2007) Climate neutral ice cream – Ben & Jerry’s ‘Fossil Fuel’. Confidential report by Ecofys in March 2007.

73 Amienyo D, Azapagic A (2016) Life cycle environmental impacts and costs of beer production and consumption in the UK. Int J Life Cycle Assess 21 (4):492–509. <https://doi.org/10.1007/s11367-016-1028-6>

74 Carlsberg (2019) <https://www.carbontrust.com/media/674493/carlsberg-group-case-study.pdf>, viewed 15.12.2019

# Supplement C

## Additional result tables

**Table C1.** Characteristics of the participants who completed randomized controlled trial (the ScenoProt intervention study). The diet groups in the 12-week intervention (n=136) were as follows: ANIMAL with the ratio of animal protein to plant protein 70:30 (n=45), 50/50 with the ratio of 50:50 (n=46), and PLANT with the ratio of 30:70 (n=44).

|  | **ANIMAL**  **(*n* = 46)** | **50%/50**  **(*n* = 46)** | **PLANT**  **(*n* = 44)** | **All**  **(*n* = 136)** |
| --- | --- | --- | --- | --- |
| Age (years) | 47.6 ± 14.5 | 47.2 ± 14.7 | 48.7 ± 14.0 | 47.8 ± 14.3 |
| Sex ^a^ |  |  |  |  |
| Female (all/total) | 37 (80) | 36 (78) | 34 (77) | 107 (79) |
| Male | 9 (20) | 10 (22) | 10 (23) | 29 (21) |
| Education ^a b^ |  |  |  |  |
| Upper secondary or less | 10 (22) | 13 (28) | 7 (16) | 30 (22) |
| Bachelor’s degree or higher | 33 (71) | 26 (57) | 35 (80) | 94 (69) |
| BMI (kg/m^2^) baseline | 24.7 ± 4.1 | 24.4 ± 3.9 | 25.2 ± 3.7 | 24.9 ± 3.9 |
| BMI (kg/m^2^) endpoint | 25.2 ± 4.2 | 24.5 ±4.1 | 25.1 ± 3.8 | 24.9 ± 4.0 |
| Systolic blood pressure (mmHg) baseline | 121.7 ± 14.5 | 119.7 ± 15.0 | 126.5 ± 16,5 | 122.3 ± 15.2 |
| Systolic blood pressure (mmHg) endpoint | 121.6 ± 14.7 | 118.8 ± 15.1 | 123.1 ± 13.0 | 120.6 ± 14.1 |
| Diastolic blood pressure (mmHg) baseline | 75.0 ± 11.2 | 74.2 ± 9.2 | 77.0 ± 9.6 | 75.1 ± 10.0 |
| Diastolic blood pressure (mmHg) endpoint | 75.3 ± 9.1 | 72.4 ± 8.5 | 75.8 ± 8.3 | 74.2 ± 8.5 |
| Non-HDL-cholesterol (mmol/l) baseline | 3.6 ± 1.0 | 3.8 ± 0.8 | 3.6 ±0.9 | 3.7 ± 0.9 |
| Non-HDL-cholesterol (mmol/l) endpoint | 3.6 ± 0.9 | 3.5 ± 0.9 | 3.3 ± 0.8 | 3.5 ± 0.9 |
| Triglycerides (mmol/l) baseline | 1.1 ± 0.5 | 1.0 ± 0.4 | 1.0 ± 0.3 | 1.0 ± 0.4 |
| Triglycerides (mmol/l) endpoint | 1.2 ± 0.5 | 1.0 ± 0.5 | 1.0 ± 0.3 | 1.0 ± 0.4 |
| Ratio of LDL/HDL cholesterol baseline | 1.9 ± 0.8 | 2.0 ± 0.7 | 1.8 ± 0.7 | 1.9 ± 0.7 |
| Ratio of LDL/HDL cholesterol endpoint | 2.0 ± 0.8 | 1.9 ± 0.7 | 1.7 ± 0.7 | 1.8 ± 0.7 |
| Ratio of PINP/CTX baseline ^c^ | 219.3 ± 89.1 | 209.9 ± 86.7 | 220.2 ± 106.8 | 216.0 ± 93.5 |
| Ratio of PINP/CTX endpoint ^c^ | 224.5 ± 76.6 | 201.1 ± 76.4 | 171.0 ± 62.9 | 199.5 ± 75.1 |
| Calcium intake (mg) baseline^d^ | 1304.8 ± 425.2 | 1148.8 ± 415.3 | 1152.3 ± 281.4 | 1192.9 ± 380.4 |
| Calcium intake (mg) endpoint | 1189.2 ± 328.9 | 817.0 ± 251.3 | 733.5 ± 166.0 | 913.9 ± 315.8 |
| Vitamin D (μg/day) baseline^d^ | 9.7 ± 5.0 | 8.2 ± 4.2 | 9.5 ± 5.5 | 9.2 ± 4.8 |
| Vitamin D (μg/day) endpoint | 8.0 ± 4.2 | 6.2 ± 3.8 | 6.1 ± 3.8 | 6.8 ± 4.0 |
| Saturated fat (g/day) baseline^d^ | 32.7 ± 10.2 | 30.2 ± 11.1 | 29.5 ± 9.8 | 30.8 ± 10.4 |
| Saturated fat (g/day) endpoint | 31.8 ± 7.8 | 24.2 ± 7.3 | 22.1 ± 6.9 | 26.3 ± 8.4 |
| Animal-sourced iron (mg/day) baseline^d^ | 4.1 ± 2.0 | 3.8 ± 2.0 | 3.8 ± 2.3 | 3.8 ± 2.1 |
| Animal-sourced iron (mg/day) endpoint | 4.3 ± 1.6 | 2.8 ± 1.2 | 1.5 ± 0.9 | 2.8 ± 1.7 |
| Fibre (g/day) baseline^d^ | 30.0 ± 12.3 | 28.4 ± 8.7 | 27.7 ± 8.4 | 28.7 ± 9.9 |
| Fibre (g/day) endpoint | 28.9 ± 13.1 | 34.7 ± 10.7 | 37.0 ± 7.0 | 33.5 ± 11.1 |

Values are means ± SDs.

^a^ Values are n (%).

^b^ For education data, total *n* = 124 (data missing for 12 subjects).

^c^ For PINP/CTX ratio n = 134 (data missing for 2 subjects).

^d^ For all baseline intakes n = 134 (data missing for 2 subjects).

**Table C2.** Spearman’s rank correlation coefficients and their *p*-values between climate impact (realised), certain health indicators, and nutrient intakes in intervention groups.

|  | ANIMAL | | 50/50 | | PLANT | |
| --- | --- | --- | --- | --- | --- | --- |
|  | r | p-value | r | p-value | r | p-value |
| BMI (kg/m^2^) | -0.197 | 0.190 | 0.128 | 0.398 | 0.1204 | 0.436 |
| Systolic blood pressure (mmHg) | 0.039 | 0.795 | -0.082 | 0.588 | -0.032 | 0.832 |
| Diastolic blood pressure (mmHg) | 0.005 | 0.974 | -0.138 | 0.361 | -0.079 | 0.606 |
| Non-HDL cholesterol (mmol/l) | -0.216 | 0.150 | 0.117 | 0.439 | -0.015 | 0.924 |
| Triglycerides (mmol/l) | -0.024 | 0.869 | -0.010 | 0.943 | -0.127 | 0.409 |
| Ratio of LDL/HDL cholesterol | -0.176 | 0.240 | 0.230 | 0.123 | -0.002 | 0.989 |
| PINP/CTX ratio | -0.056 | 0.715 | 0.316 | 0.035 | 0.012 | 0.942 |
| Total NOC (pmol/mg) | 0.007 | 0.965 | 0.007 | 0.960 | -0.006 | 0.966 |
| Haem NOC (pmol/mg) | -0.006 | 0.968 | 0.059 | 0.700 | -0.098 | 0.523 |
| Glucose (mmol/l) | -0.234 | 0.118 | 0.189 | 0.206 | 0.188 | 0.220 |
| Insulin (mU/l) | -0.285 | 0.058 | -0.242 | 0.104 | 0.104 | 0.506 |
| HOMA2-IR indes | 0.296 | 0.047 | 0.203 | 0.173 | -0.117 | 0.454 |
| Saturated fat (g/day) | 0.259 | 0.088 | 0.237 | 0.111 | 0.246 | 0.106 |
| Vitamin D (µg/day) | 0.076 | 0.615 | -0.201 | 0.179 | 0.184 | 0.231 |
| Cacium (mg/day) | 0.375 | 0.010 | 0.093 | 0.537 | 0.439 | 0.002 |
| Animal-sourced iron (mg/day) | 0.592 | <0.001 | 0.705 | <0.001 | 0.584 | <0.001 |
| Fibre (g/day) | 0.367 | 0.012 | 0.325 | 0.027 | 0.098 | 0.526 |
|  |  |  |  |  |  |  |

**Table C3.** Spearman’s rank correlation coefficients and their *p*-values between climate impact (per 2,000 kcal), certain health indicators, and nutrient intakes in intervention groups.

|  | ANIMAL | | 50/50 | | PLANT | |
| --- | --- | --- | --- | --- | --- | --- |
|  | r | p-value | r | p-value | r | p-value |
| BMI (kg/m^2^) | -0.136 | 0.364 | 0.132 | 0.380 | 0.123 | 0.424 |
| Systolic blood pressure (mmHg) | 0.157 | 0.294 | -0.241 | 0.105 | 0.078 | 0.611 |
| Diastolic blood pressure (mmHg) | 0.006 | 0.964 | -0.226 | 0.129 | -0.048 | 0.754 |
| Non-HDL cholesterol (mmol/l) | -0.055 | 0.716 | 0.307 | 0.038 | 0.019 | 0.902 |
| Triglycerides (mmol/l) | 0.024 | 0.870 | 0.044 | 0.768 | -0.180 | 0.241 |
| PINP/CTX ratio | -0.070 | 0.647 | 0.176 | 0.248 | 0.213 | 0.175 |
| Total NOC (pmol/mg) | 0.035 | 0.815 | 0.180 | 0.235 | 0.217 | 0.156 |
| Haem NOC (pmol/mg) | 0.015 | 0.920 | 0.200 | 0.187 | 0.216 | 0.158 |
| Glucose (mmol/l) | -0.052 | 0.730 | 0.218 | 0.144 | 0.167 | 0.276 |
| Insulin (mU/l) | -0.237 | 0.115 | -0.023 | 0.877 | 0.054 | 0.728 |
| Homa2-IR index | 0.242 | 0.108 | -0.017 | 0.907 | -0.051 | 0.745 |
| LDL/HDL ratio | -0.201 | 0.180 | 0.333 | 0.023 | -0.094 | 0.543 |
| Saturated fat (g/day) | -0.056 | 0.710 | -0.028 | 0.850 | -0.179 | 0.242 |
| Vitamin D (µg/day) | -0.075 | 0.618 | -0.307 | 0.037 | -0.041 | 0.787 |
| Cacium (mg/day) | 0.078 | 0.604 | -0.183 | 0.221 | 0.056 | 0.713 |
| Animal-sourced iron (mg/day) | 0.333 | 0.023 | 0.386 | 0.007 | 0.517 | <0.001 |
| Fibre (g/day) | -0.054 | 0.723 | -0.136 | 0.368 | -0.441 | 0.003 |
